# [2 + 1] Cycloaddition reactions of fullerene C_60_ based on diazo compounds

**DOI:** 10.3762/bjoc.17.55

**Published:** 2021-03-05

**Authors:** Yuliya N Biglova

**Affiliations:** 1 Department of Chemistry, Bashkir State University, 450076, Ufa, Russian Federation

**Keywords:** [2 + 1]-cycloaddition processes, analogues of [60]PCBM, diazo compounds, mechanisms and optimal conditions of cyclopropanation, methanofullerenes C_60_

## Abstract

The most common variant of fullerene core functionalization is the [2 + 1] cycloaddition process. Of these, reactions leading to methanofullerenes are the most promising. They are synthesized in two main reactions: nucleophilic cyclopropanation according to the Bingel method and thermal addition of diazo compounds. This present review summarizes the material on the synthesis of monofunctionalized methanofullerenes – analogues of [60]PCBM – based on various diazo compounds. The main cyclopropanating agents for the synthesis of monosubstituted methanofullerenes, the optimal conditions and the mechanism of the [2 + 1] cycloaddition, as well as the practical application of the target products are analyzed.

## Introduction

Taking into consideration that all life on Earth is of organic origin. with carbon as its basic element (20% by weight of every living organism, and the fourth most abundant element in the universe), the discovery in 1985 of its new allotropic modification, fullerene, has resulted in an avalanche-like expansion of studies in this area [1]. The exceptional physicochemical properties of the fullerene family and, above all, its main representative, C_60_, predetermine the broad practical interest in the use of these compounds. This is quite understandable: compounds with unique optical [2–4], electrophysical [5–7], mechanical [8–10], tribological [11–13], sorption [14–17], and biological properties [18–20], and even new dyes and catalysts [21–25], have already been discovered among functionalized fullerenes.

Bearing in mind the fact that numerous fullerene derivatives with a wide range of biological activity have been synthesized to date, the idea of using functionalized fullerenes in the field of medical chemistry, especially for the treatment of socially significant diseases, is fruitful.

The design of biologically active compounds based on C_60_ is determined, on the one hand, by the ability of its shell to serve as a transportation unit for pharmacophore groups, and on the other by the need to identify the capability of the fullerene core itself (depending on the nature of the attached fragment) to exhibit inherent bioactive properties in a living organism. The efforts of chemists in the design and synthesis of organofunctionalized C_60_ derivatives for medicine are primarily focused on the creation of water-soluble compounds [18,26–27]. Fullerene-containing compounds with antiviral (various forms of influenza, herpes, HIV) [28–30], antibacterial [31–33], and anticancer activity [34–35] have been found among them. An important approach in this area involves the synthesis of complex- and covalent-bound fullerene derivatives with biologically active molecules that already find practical use. It has been found that complexation improves the transportation of a drug and prolongs its effect, and a synergistic effect is observed in some cases [36].

The situation with covalent binding in fullerene–drug conjugates is different. C_60_ is an ideal partner for drug lipophilization by conjugation [37]. Highly efficient fullerene conjugates with chemotherapeutic agents have been synthesized and patented; moreover, the biological activity profile is often preserved, but in some cases it can also change since a molecular structure with a fundamentally new architecture appears [38].

The neuroprotective activity of fullerene derivatives may not be ignored either. Encouraging results of their use for the treatment of neurodegenerative pathologies (Alzheimer's and Parkinson's diseases and other diseases accompanied by cognitive impairment) leading to dementia have been demonstrated [39–40]. Moreover, it is noted that the almost nonexistent toxicity of the C_60_ derivatives allows to use them in living systems without negative consequences.

Studies on the use of C_60_ derivatives in micro- and nanoelectronics are also important and very promising. For example, fullerene-containing materials are used as electron-withdrawing components and buffer layers in the development and design of photoconductors [41–42], supercapacitors [43–44], field effect transistors (FET) [45–46], organic light-emitting diodes (OLEDs) [47–48] and organic or hybrid solar cells [49–50]. In view of the globalization of energy problems, the issues of switching to alternative energy sources are becoming increasingly relevant. Solar energy, like the majority of renewable energy sources, is environmentally friendly and has almost no negative impact on the environment.

Catalysis is the next area that is gaining an ever-increasing practical focus in the studies on the use of C_60_ and fullerene-containing materials [51]. A comparative analysis of the activity of fullerene-containing compounds and noble metals as dehydrogenation catalysts allows to consider the former as alternatives that favorably differ in cost. However, the efficiency is similar to the functional analogs of catalytic additives based on noble metals [52]. The presence of metal–fullerene chemical bonding results in catalysts that reliably provide high process selectivity due to the simultaneous adsorption of the substrate on two sites, namely a metal and a fullerene [53]. This allows to level out some negative features that are characteristic of heterogeneous catalysts. Thus, in dehydrogenation processes, catalytic amounts of a coordinated fullerene in metal fullerides act as a hydrogen acceptor in reactions with compounds containing activated C–H bonds [54].

An ample body of data on the chemistry of fullerenes has been accumulated to date. It is covered in a number of monographs [55–58] and reviews [59–75]. This review, in which we discuss the primary functionalization of the fullerene core with diazo compounds to synthesize solely monosubstituted carbochain products, covers the main achievements of organic chemistry over the past 20 years in the field of [2 + 1] cycloadditions to fullerene.

## Review

### [2 + 1] Cycloaddition to C_60_ to give methanofullerenes

Scientists are currently focusing on methods for the functionalization of the fullerene core that provide a high selectivity and yield. The electron-deficient polyene C_60_ readily undergoes radical addition, nucleophilic addition, and cycloaddition processes. Therein, the decrease in the strain in the fullerene core should be considered as the driving force of the reactivity. The [2 + *n*] cycloaddition reactions, where *n* varies from 1 to 4, are the most promising in the functionalization of the fullerene sphere. The [2 + 1] cycloaddition is the most popular variant among synthetic organic chemists as it allows to obtain methanofullerenes, fullerenoaziridines, or fullerenooxyranes. These reactions can involve, for example, the addition of stabilized carbanions, carbenes, and nitrenes and can involve various reaction mechanisms. While in the early years of intense research on reactions of cycloaddition to C_60_, the search for diverse addends for various cycloaddition variants as well as the determination of the main regularities of these processes were of primary interest, but the trends of studies recently changed. They are now focused on the targeted synthesis of fullerene derivatives with specific useful properties, creation of fundamentally new reagents for cycloaddition, and detailed studies on the multiaddition processes and the characteristics of polycyclic adducts.

**Scheme 1:**
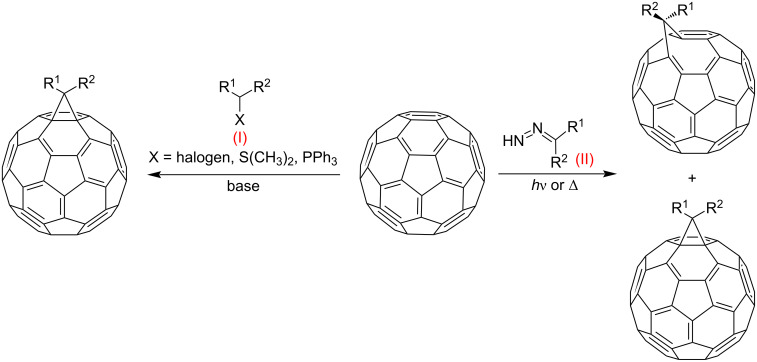
Methods for the synthesis of methanofullerenes C_60_.

The majority of researchers developing methods for C_60_ functionalization prefer [2 + 1] cycloaddition processes, among which reactions leading to methanofullerenes are widely used. Two main synthetic methods for the production of methanofullerenes can be distinguished (Scheme 1): nucleophilic cyclopropanation with stabilized carbanions that occurs by the addition–cleavage mechanism – the Bingel reaction (I) and addition of diazo compounds at elevated temperatures with subsequent release of N_2_ (II). The cyclopropanation of C_60_ with stabilized α-halocarbanions, i.e., the Bingel reaction, is the most efficient way to synthesize methanofullerenes [76]. It is almost versatile; in the presence of a reactive methylene function in halo-substituted substrates and with facilitation of bases, methanofullerenes with various structures are formed rather smoothly. It is believed that nucleophilic addition of an α-halocarbanion to C_60_ occurs initially, followed by intramolecular substitution of the halogen atom by an anionic center (which is generated on the fullerene sphere). In the initial variant, cyclopropanation occurs upon treatment of C_60_ with 2-bromomalonic ester in the presence of a base; the reaction is completed quickly, and the yields are good in most cases. However, this technique involves an additional stage, viz, it becomes necessary to preliminarily halogenate the cyclopropanating agent. As a rule, a hardly separable mixture of mono- and dihalo-containing products is formed.

The Hirsch-modified Bingel method makes it possible to isolate methanofullerenes by a one-pot reaction using simultaneously CBr_4_ or I_2_, 1,8-diazabicyclo[5.4.0]undec-7-ene (DBU) or NaH as the base, and a malonate-derived substrate. The adduct is synthesized in a slightly higher yield by direct treatment of fullerene with malonates.

**Figure 1:**
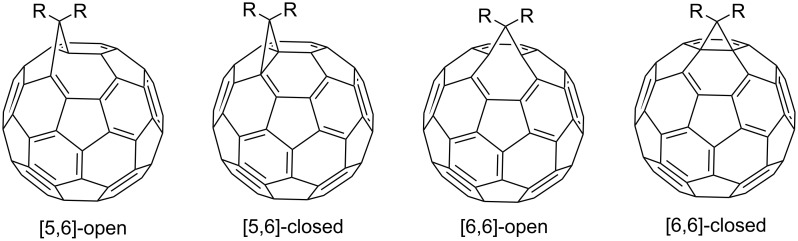
Four theoretically possible isomeric methanofullerenes C_60_.

As noted above, a decrease in the strain of the fullerene core is the driving force of its reactivity. According to the theory, monosubstituted fullerenes can exist as the four isomers shown in Figure 1: [5,6]-closed, [5,6]-open, [6,6]-closed, and [6,6]-open. Only [5,6]-open and [6,6]-closed have been isolated experimentally. This is explained by the preservation of energy-favorable levels in these isomers [77–78]. It has been shown by calculations that two of the four possible structures mentioned above are thermodynamically stable: [6,6]-isomers of methanofullerenes, which are characterized by a closed transannular bond (π-homoaromatic structure), as well as [5,6]-isomers, which have an open transannular bond (σ-homoaromatic structure) [79]. It was found that, as a rule, the [5,6]-open adducts formed initially can undergo rearrangement into [6,6]-closed isomers that are more thermodynamically stable.

An alternative variant of [2 + 1] cycloadditions to the fullerene frame involves the thermal addition of diazo compounds (the well-known carbene precursors), followed by N_2_ elimination. However, it occurs much more difficultly for diazo compounds than with singlet carbenes. This is due to the formation of a mixture of [5,6]-closed and [6,6]-open isomeric cyclic adducts. This phenomenon can be due to a number of reasons. First, two cyclopropanation mechanisms are equally likely in the thermal reaction of diazo compounds with C_60_ fullerene: a) preliminary carbene formation due to the thermal decomposition of diazo compounds, followed by synchronous addition to the double [6,6]-bond in C_60_ or b) possible formation of both isomers as a result of 1,3-dipolar cycloaddition of the diazo compound to the fullerene, followed by elimination of molecular nitrogen from the pyrazoline intermediate. Second, the rearrangement of [5,6]-open isomers into thermodynamically more stable [6,6]-closed ones is plausible. In fact, it has been proved convincingly [79] that a series of [5,6]-open fulleroids with stabilizing substituents in the methane bridge are rearranged into [6,6]-closed fullerenes, both by a photochemical process of zero-order kinetics and by a high-energy monomolecular route. The latter involves the closure into a [5,6]-closed fullerene, which is then regrouped into a [6,6]-closed fullerene via a biradical intermediate (Scheme 1). Nevertheless, examples of the synthesis of both individual isomeric cycloadducts and mixtures of isomeric fullerene and fulleroid structures upon addition of diazo compounds to C_60_ have appeared in recent years. Though the reaction has to be performed with heating, the yield of a functionalized C_60_ is much higher than in the nucleophilic cyclopropanation with stabilized carbanions. This variant for synthesizing methanofullerenes is also attractive because the variability of the substituents in the cyclopropane moiety is very high.

### Cyclopropanation of C_60_ with diazo compounds

**Scheme 2:**
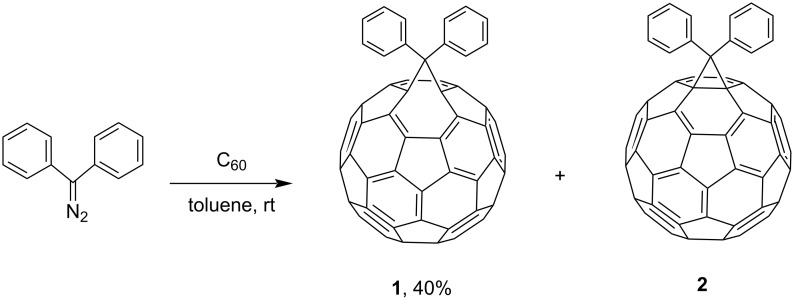
Synthesis of diphenyl-substituted fulleroids **1** and **2**.

At the end of 1991, Suzuki [80] reported the first fullerene-containing derivative obtained by nucleophilic addition of diphenyldiazomethane to C_60_. The reaction of C_60_ with more than one equivalent of diphenyldiazomethane in toluene at room temperature for ca. 1 hour gives a monoadduct, i.e., a diphenyl derivative, in a yield of about 40%, with the characterized preferred structure **2** (Scheme 2).

**Scheme 3:**
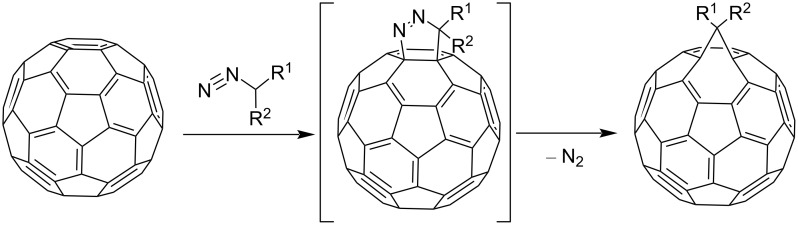
The reaction mechanism of a family of fulleroids by cyclopropanation of C_60_ with diazo compounds.

As a result of diazo addition to the fullerene core, the loss of molecular nitrogen occurs according to Scheme 3:

Subsequently, it was shown in papers on the chemical reactions of C_60_ that this reaction allows substituents to be present in phenyl rings, which implies a general applicability for the development of functionalized fullerene derivatives.

**Scheme 4:**
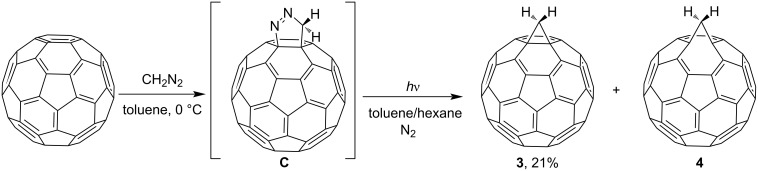
Synthesis of 1,2-methanobuckminsterfullerene **3** and related fulleroid **4**.

Treatment of C_60_ with an ethereal solution of diazomethane in toluene at 0 °C resulted in pyrazoline derivative **C** [81]. The adduct isolated chromatographically was exposed to ultraviolet (UV) irradiation for 25 min at 25 °C in a quartz tube. The resulting isomers **3** and **4** were separated by reversed-phase high-performance liquid chromatography (HPLC) (methanol/toluene as the eluent) to isolate a reddish-brown powder of methanofullerene **3** in 21% yield (Scheme 4).

**Scheme 5:**
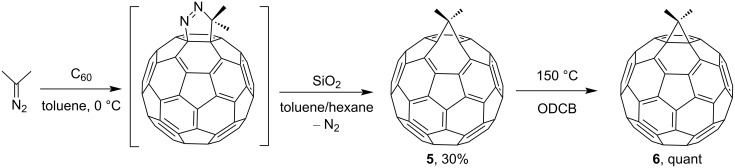
Synthesis of dimethylmethanofullerene **6** from dimethyldiazomethane via the formation of annulene **5**. ODCB = orthodichlorobenzene.

Similar to photolysis, the thermal rearrangement of kinetic products gives [6,6]-closed isomers that are thermodynamically more stable. After some time, the team of scientists mentioned the synthesis of dimethylmethanofullerene **6** from dimethyldiazomethane via the formation of annulene **5** according to Scheme 5 [82]. The reactions of C_60_ with other diazo derivatives also result in mixtures of various isomers. The ratio between the [5,6]-open fulleroid type and [6,6]-open methanofullerene in the reaction mixture after the loss of H_2_ depends on the diazo compound used for the cycloaddition. According to ^13^C NMR data, the [6,5]-bridged compound **5** has an open transannular bond, while the [6,6]-bridged compound **6** has a closed *trans*-ring bond.

**Scheme 6:**
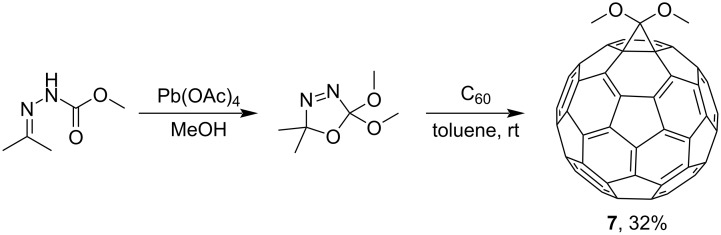
Synthesis of dimethoxymethanofullerene **7**.

**Scheme 7:**
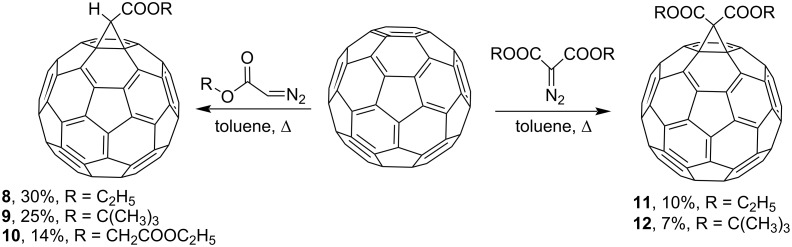
Synthesis of fullerene monoesters and symmetric diesters **8**–**10** and **11**–**12**, respectively.

A synthetically interesting building block, dimethoxymethanofullerene **7**, was obtained by Diederich and Isaacs according to Scheme 6 [83]:

It was demonstrated that electron-withdrawing diazoacetates **8**–**10** and symmetric diazomalonates **11** and **12** can be involved in the reaction with C_60_ to give the corresponding methanofullerenes (Scheme 7) [77,83–84]. Later, the C_60_ adduct **8** and diethyl dicarboxymethanofullerene **11** were synthesized in Rh_2_(OAc)_4_-catalyzed reactions. Using Rh_2_(OAc)_4_, the selectivity of the process was improved, and the yield of the target products slightly increased to 42 and 32%, respectively [85].

**Scheme 8:**
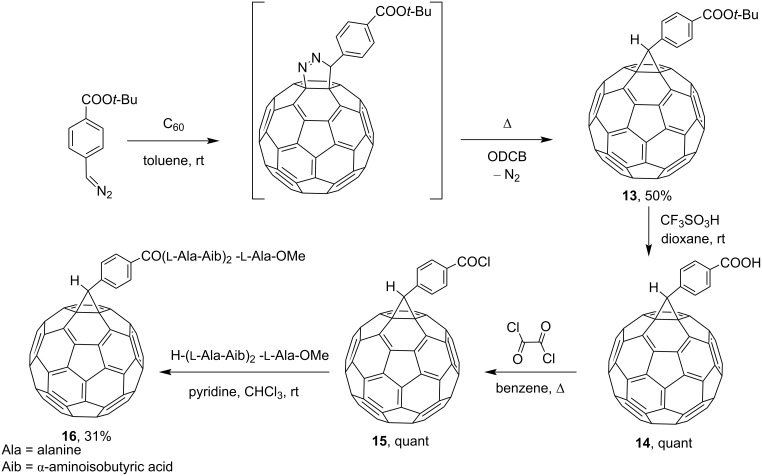
The synthetic route to the first fullerene–peptide conjugate **16**.

The ability of derivatives to effectively preserve the main characteristics of C_60_ stimulated a great interest in the application of fullerenes in medical chemistry. Of the fullerene-based biologically significant molecules, amino acid derivatives are of particular interest. In fact, Prato and co-workers synthesized and characterized the first peptide with a covalently attached fullerene molecule [86]. To this end, adduct **13** was obtained from 4*-*(*tert*-butoxycarbonyl)phenyldiazomethane and fullerene. Subsequent acid hydrolysis of the protective ester group quantitatively gave a derivative of carboxylic acid **14**, a versatile synthon for the synthesis of amphiphilic C_60_ derivatives. Treatment of **14** with oxalyl chloride gives reactive acid chloride **15**. Acylation of the presynthesized (ʟ-Ala-Aib)_2_-ʟ-Ala-OMe peptide with compound **15** gives the expected fullerene–peptide conjugate **16** (Scheme 8).

**Scheme 9:**
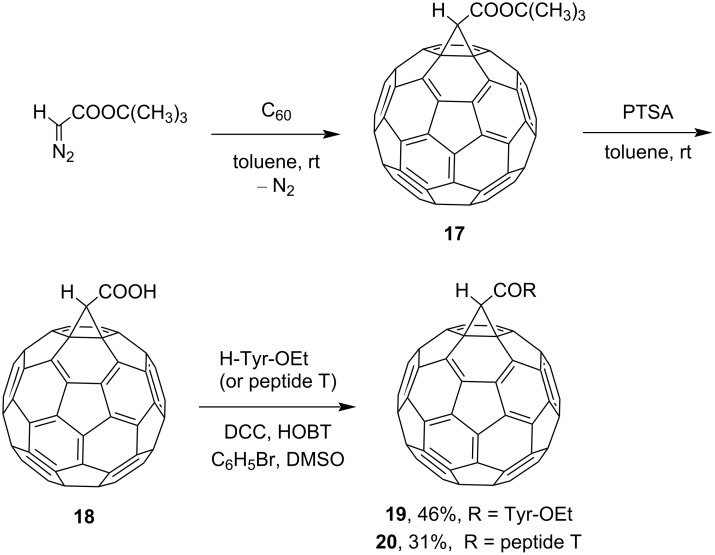
Synthesis of bioactive fullerene peptides **19** and **20**.

**Scheme 10:**
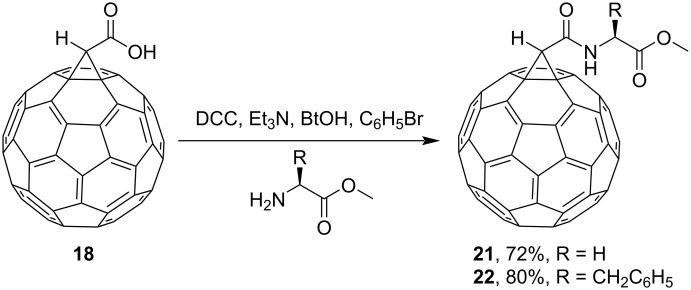
Synthesis of amino acid–fullerene derivatives **21** and **22**. (BtOH is 1*H*-benzotriazol-1-ol.)

A somewhat modified version for the synthesis of peptidofullerenes is given in Scheme 9 [87]. On this pathway for the synthesis of conjugates **19** and **20**, acid **18** is cross-linked with a peptide component using a milder carbodiimide method via the starting methanofullerene **17**. Similar to Scheme 8, fullerene adducts **21** and **22** with glycine and phenylalanine, respectively, were synthesized (Scheme 10) [83].

**Scheme 11:**
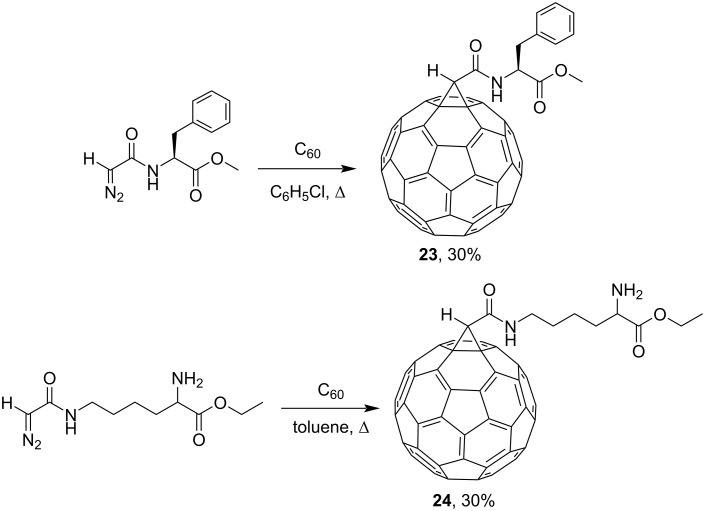
Synthesis of fullerenylpeptides **23** and **24**.

As an alternative, a more efficient direct route for synthesizing fullerenylpeptides **23** [83] and **24** [88] by prolonged thermolysis of various diazoamides in the presence of C_60_ was developed (Scheme 11).

**Scheme 12:**
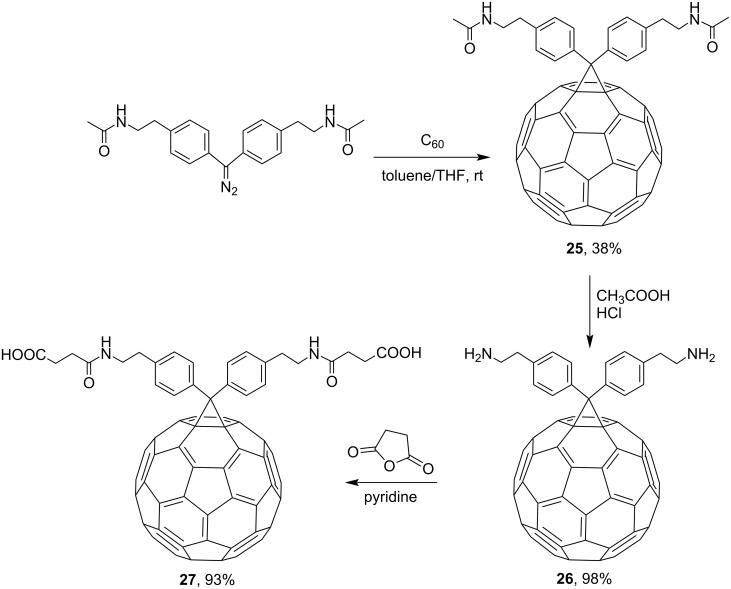
The synthetic route to the water-soluble biologically active fullerene–peptide derivative **27**.

For conjugation, some scientists successfully used mono- and diphenyldiazomethanes, available reagents carrying substituents on the phenyl rings. For example, covalent attachment of C_60_ to peptides is performed through a phenyl spacer, as it was demonstrated in a synthesis of fulleride **27** [89]. The latter compound was designed specifically for suppressing a HIV enzyme. The reaction of 4,4'-bis(*N-*acetyl-2-aminoethyl)diphenyldiazomethane with C_60_ in toluene gave adduct **25** that was isolated in 38% yield in the first stage. After that, it was quantitatively converted into primary diamine compound **26** in an acid medium. Upon addition of succinic anhydride in dry pyridine, conversion to water-soluble biologically active fullerene–peptide derivative **27** occurs (Scheme 12).

**Figure 2:**
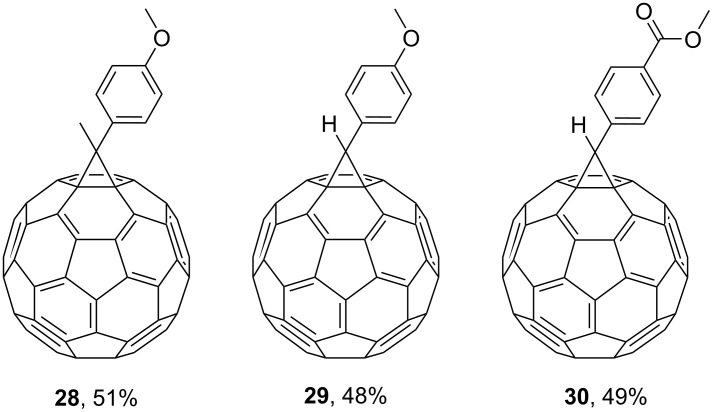
Structures of monophenyldiazomethanes **28**–**30**.

**Figure 3:**
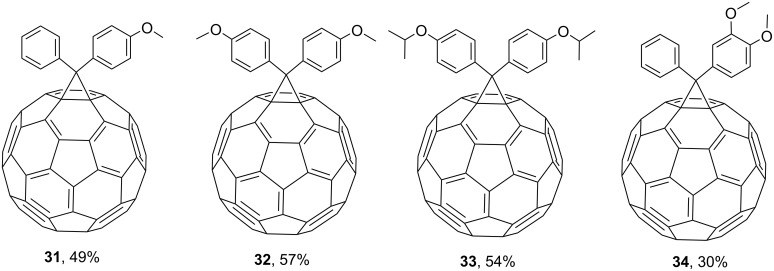
Structures of diphenyldiazomethanes **31**–**34**.

Reactions of monophenyldiazomethanes with C_60_ were used in the syntheses of adducts **28**, **29** [90], and **30** (Figure 2) [91]. Moreover, diphenyldiazomethanes were used for synthesizing **31** [79], **32**, **33** [90], and **34** (Figure 3) [92]. In all instances, a mixture of isomeric adducts is formed in a toluene solution. Heating the mixture at the solvent boiling point results in the single target product.

**Figure 4:**
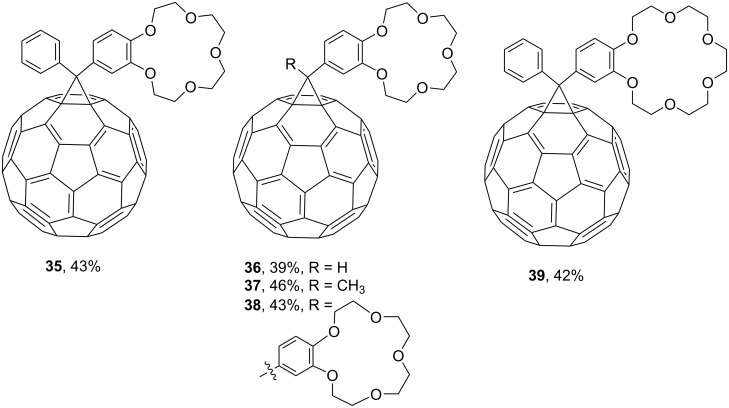
Conjugates of benzocrown ethers with phenylmethanofullerenes **35**–**39**.

The reaction of C_60_ with diazoesters at room temperature gives adducts of benzo-15-crown-5 with phenylmethanofullerenes **35** [92] and **36**–**38** (Figure 4) [93]. A synthesis based on benzo-18-crown-6 to give **39** showed that attachment of the crown ether to the surfaces of fullerene molecules makes it easier to detect them by mass spectrometry [94]. This method was used to incorporate benzocrown ether residues in order to study the complexing properties of new fullerene derivatives.

**Figure 5:**
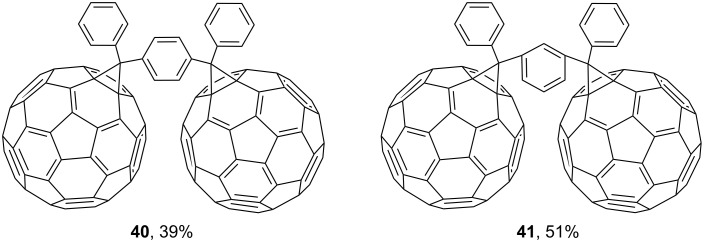
Structures of *para*-phenylenebis(phenylfulleroid) **40** and *meta*-phenylenebis(phenylfulleroid) **41**.

An interesting approach was developed [95] for the synthesis of diphenyl-substituted fulleroids **40** and **41** as a two-section precursor of “pearl necklace” polymers with a C_60_ core in the main chain (Figure 5). However, the adducts were poorly soluble in the majority of organic solvents and only partially soluble in carbon disulfide, which is expected to ultimately lead to similar insoluble hard-to-recover polymers. It is believed [95] that the best solution to the problem seems to involve the incorporation of solubilizing groups into the structure.

**Scheme 13:**
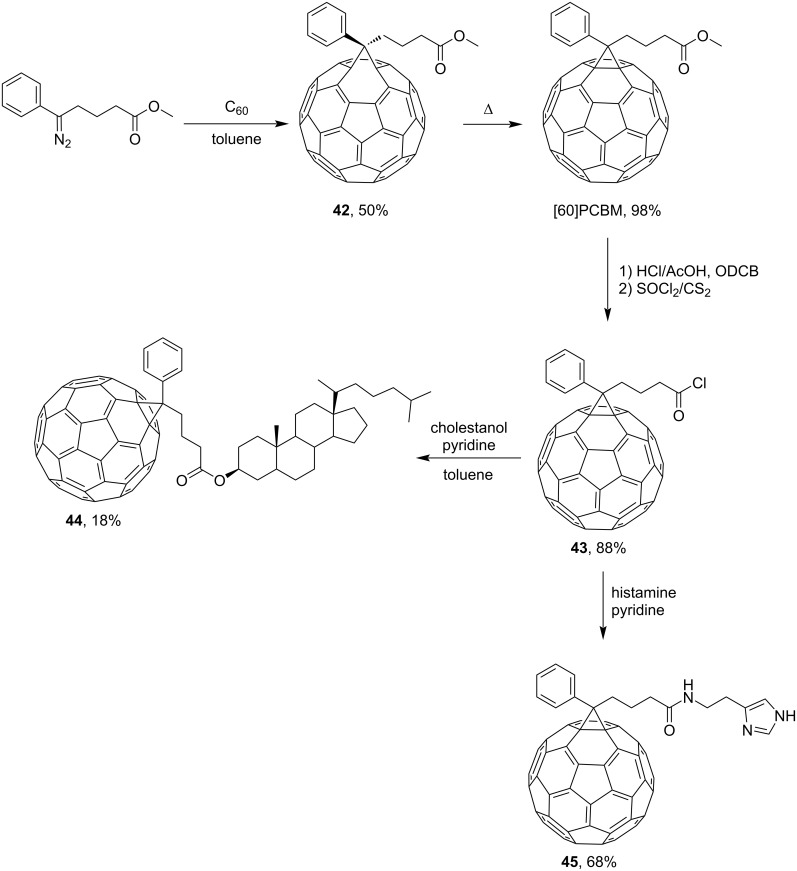
Serial synthesis of monophenylmethanofullerenes.

Of the series of monophenylmethanofullerenes, the practically important so-called [6,6]-phenyl-C_61_-butyric acid methyl ester [60]PCBM synthesized by Hummelen, Wudl, and co-workers is most prominent (Scheme 13) [96]. The authors modified [60]PCBM via acid chloride **43** to give derivatives of cholesterol **44** and histamine **45**.

**Scheme 14:**
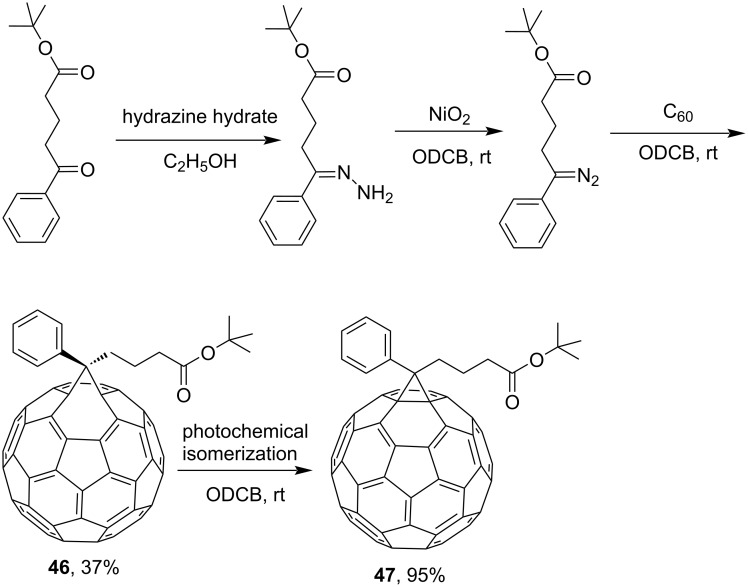
Four-step synthesis of the [6,6]-*tert*-butyl phenyl-C_61_-butyrate **47**.

Scientific literature [97] reports a three-step continuous synthesis of [6,6]-*tert*-butyl phenyl-C_61_-butyrate **47** based on *tert*-butyl 4-benzoylbutyrate hydrazone in a microstructured flow reactor that eliminates the need for stage-by-stage isolation of intermediate products and is environmentally friendly (Scheme 14).

**Scheme 15:**
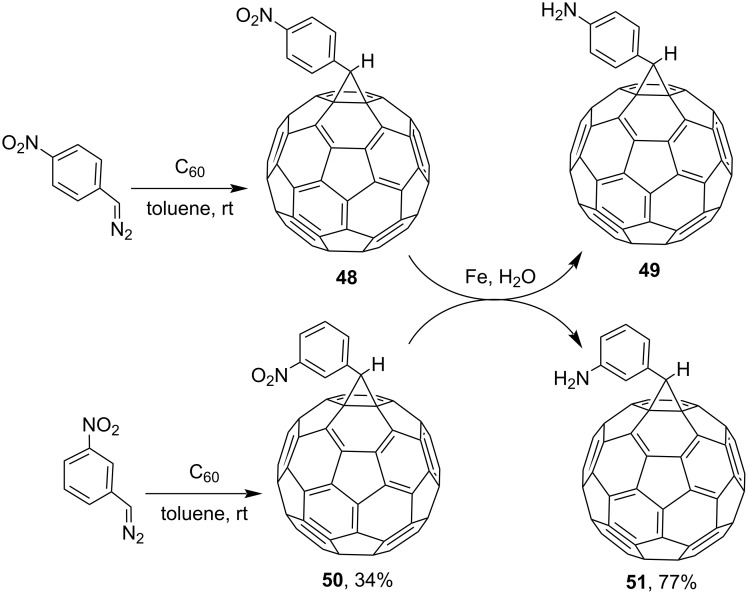
Synthesis of aminophenylfullerenes **49** and **51** based on nitrophenylfullerenes **48** and **50**.

**Scheme 16:**
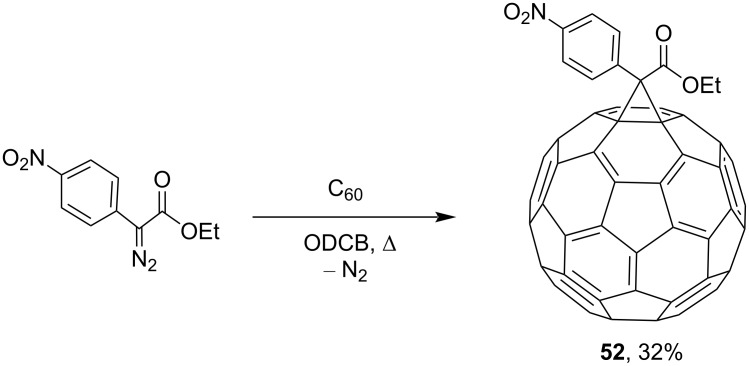
Synthesis of conjugate **52** of C_60_ with ethyl diazo(4-nitrophenyl)acetate.

Researchers obtained various nitrofullerene derivatives, viz, nitrophenylfullerenes **48** and **50**, reduction of which gave the corresponding aminophenylfullerenes **49** and **51** (Scheme 15) [98–99]. C_60_ adducts were doped with tetrakis(dimethylamino)ethylene (TDAE) and cobaltocene (Cp_2_Co) in order to create charge-transfer complexes with the purpose of searching for efficient magnetic materials based on fullerenes. It can be seen in Scheme 16 that methanofullerene **52** was obtained from the initial, also *para*-substituted, ethyl diazo(4-nitrophenyl)acetate in 32% yield [100].

**Scheme 17:**
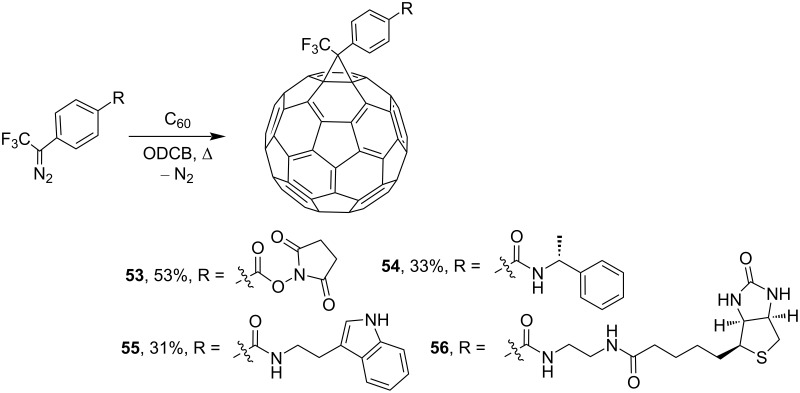
Synthesis of fluoride-containing phenylmethanofullerenes **53**–**56**.

Photolysis of derivatives of *para*-substituted 3-fluoromethyl-3-phenyldiazirones in the presence of C_60_ gave phenylmethanofullerenes **53**–**56** in acceptable yields (Scheme 17) [101]. The electrochemical properties of the products were characterized by cyclic voltammetry and differential pulse voltammetry.

**Scheme 18:**
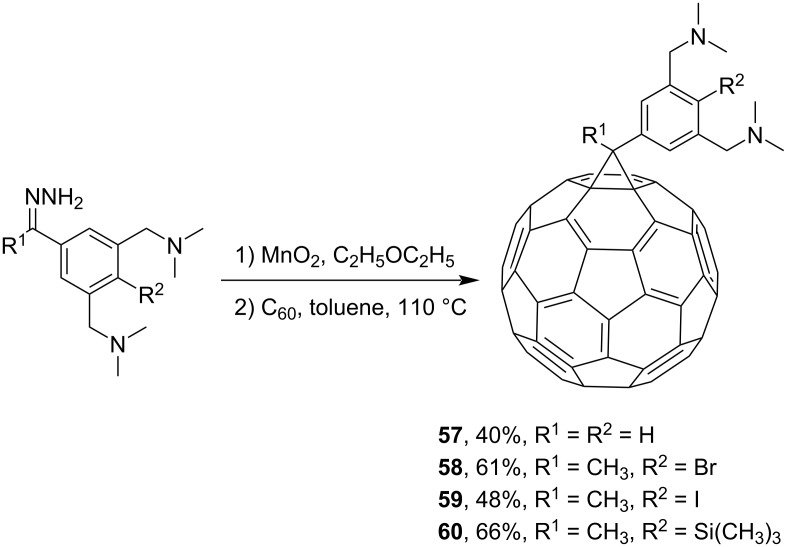
Synthesis of “bucky ligands” **57**–**60**.

One of the approaches for in situ generation of unstable diazo compounds involves the use of stable hydrazones, followed by their oxidation with MnO_2_. For example, the scientists led by van Koten and co-workers synthesized a group of methanofullerenes with monoanionic terdentate diaminoaryl pincer ligands **57**, **58** [102], **59** [103], and **60** [104], or the so-called “bucky ligands” (Scheme 18).

**Scheme 19:**
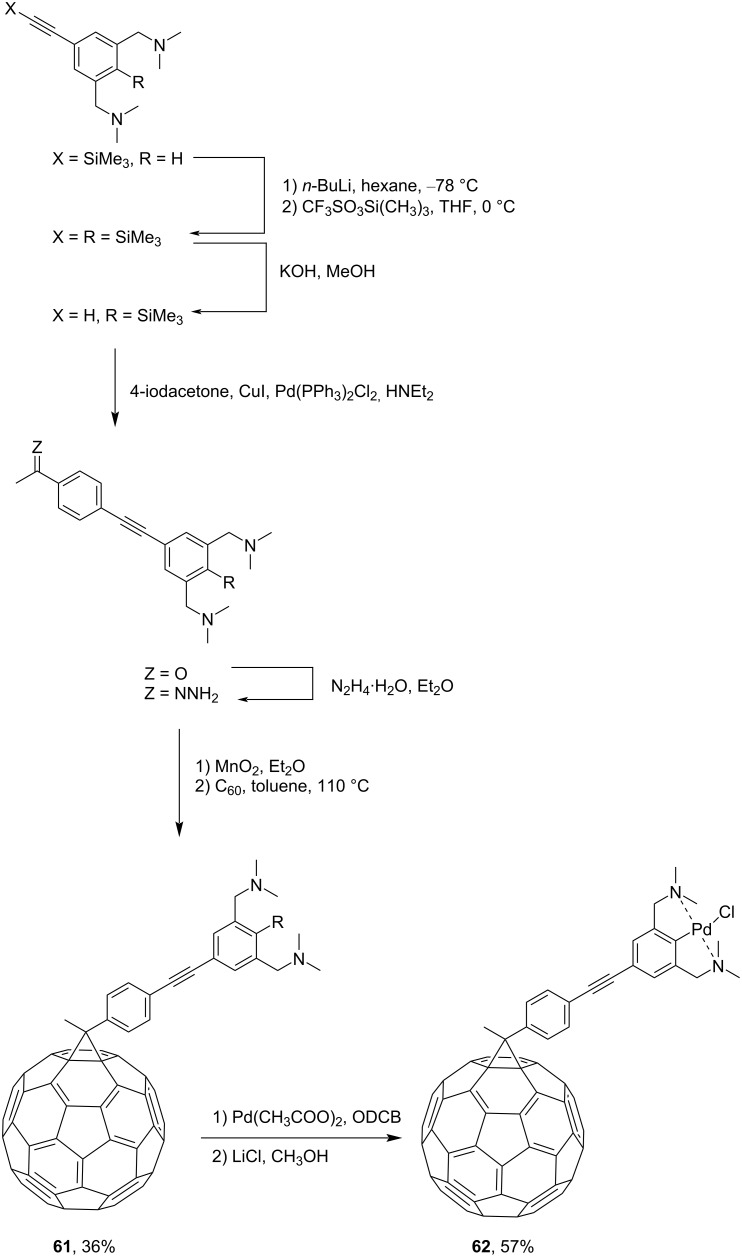
The synthetic route to methanofullerene-based palladium–bisaminoaryl complex **62**.

**Figure 6:**
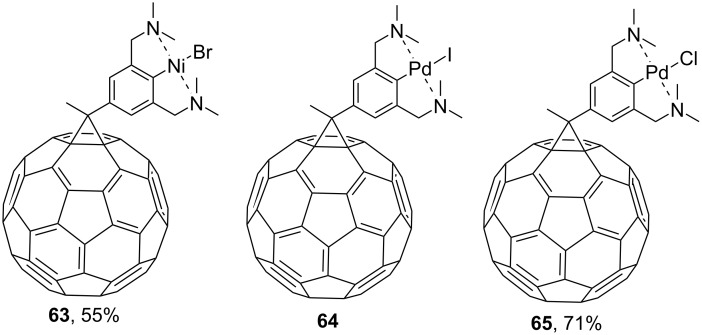
Structures of complexes **63**–**65**.

**Scheme 20:**
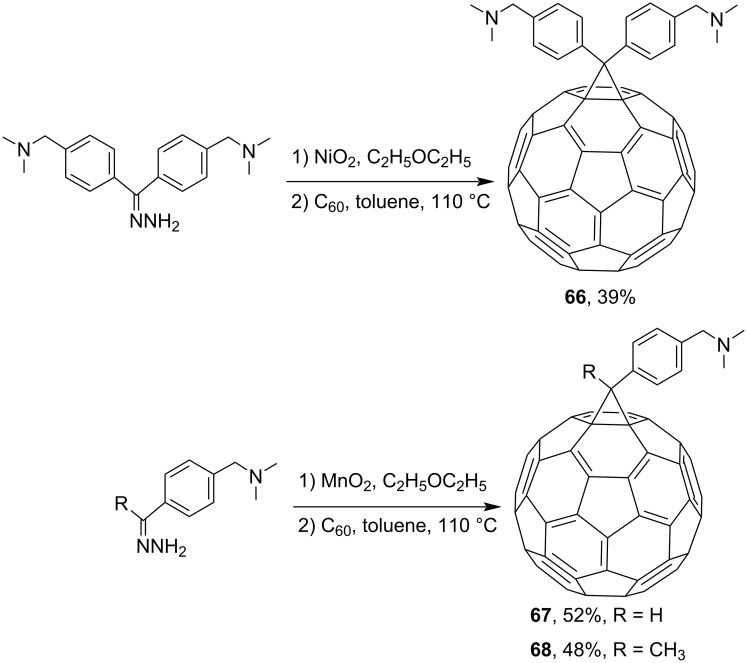
Synthesis of N*-*containing fullerene ligands **66**–**68**.

In the development of new organometallic C_60_ compounds that can be used as homogeneous catalysts, new blocks **61** and **62**, cross-conjugated through cyclopropane, were synthesized (Scheme 19) [104]. Attaching such an electron-enriched metal-containing system to C_60_ can result in push–pull blocks with new electronic and photochemical properties, as demonstrated by complexes **63**, **64** [103], and **65** [104] in Figure 6. The syntheses of new types of *N-*containing fullerene ligands for metal complexes **66**–**68** are illustrated in Scheme 20 [105].

**Scheme 21:**
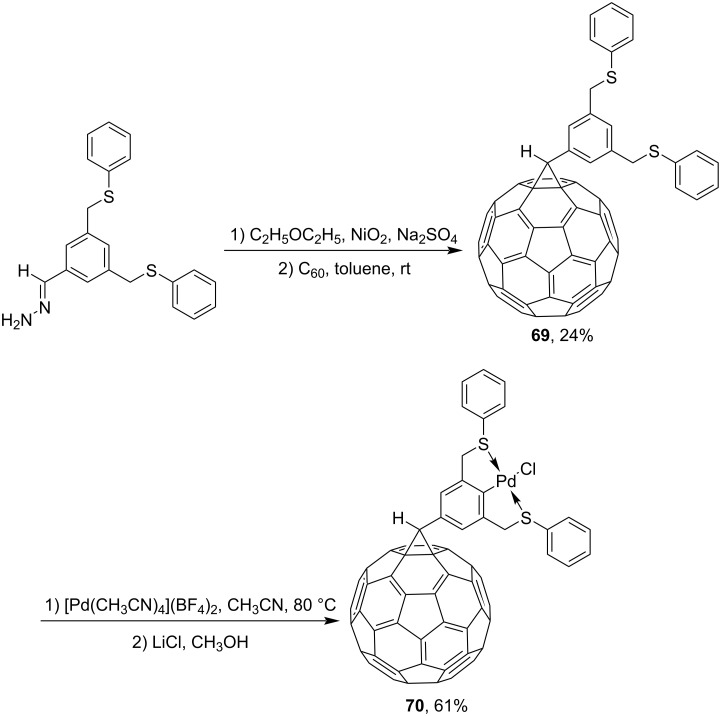
Synthesis of C_60_-attached SCS pincer–palladium(II) complex **70**.

A methanofullerene variety **69** with a pincer ligand containing a disulfidoaryl moiety was synthesized from 3,5-bis(phenylsulfidomethyl)benzaldehyde hydrazone. In the presence of [Pd(CH_3_CN)_4_](BF_4_)_2_, the latter gives the corresponding palladium complex **70** (Scheme 21) [106].

**Scheme 22:**
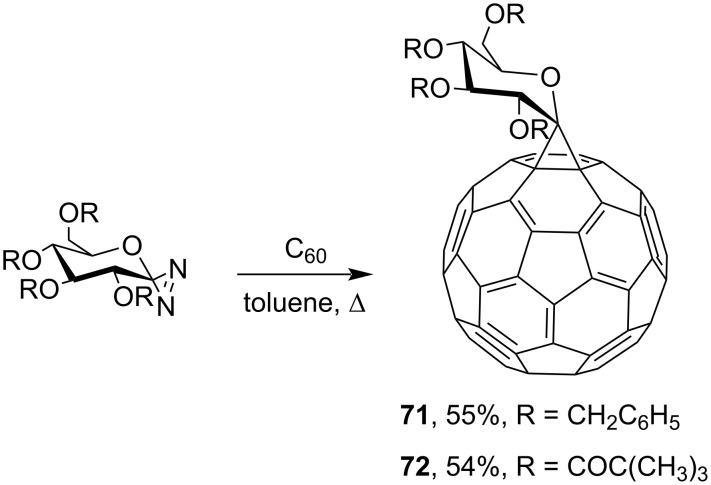
Synthesis of spiro-linked C-glycosides of fullerenes **71** and **72**.

A series of spiromethanofullerenes was synthesized using various diazo derivatives. For example, compounds spiro-bound with glycosides are the first chiral and enantiomerically pure derivatives of fullerenes **71** and **72** (Scheme 22) [107]. It should be noted that though a considerable amount of information on the synthesis of fulleropyrazolines is available, their decomposition has been studied insufficiently. The factors determining the relative stability of monoadducts obtained in reactions of C_60_ with diazo compounds also remain an open issue.

**Figure 7:**
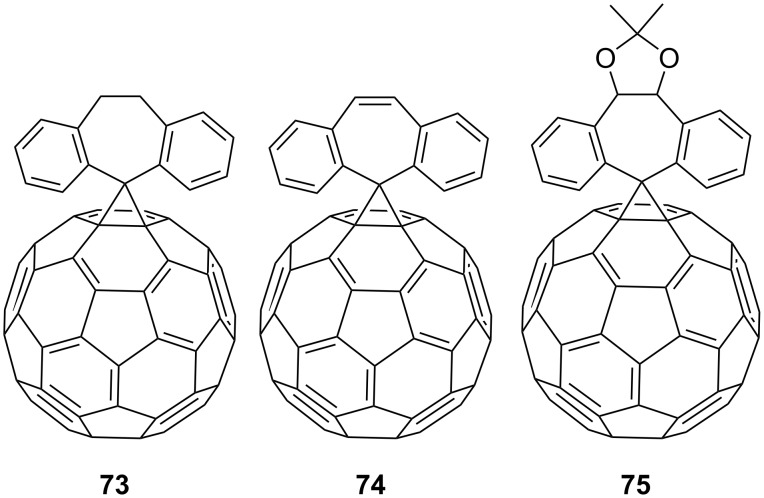
Structures of spiromethanofullerenes **73–75**.

**Scheme 23:**
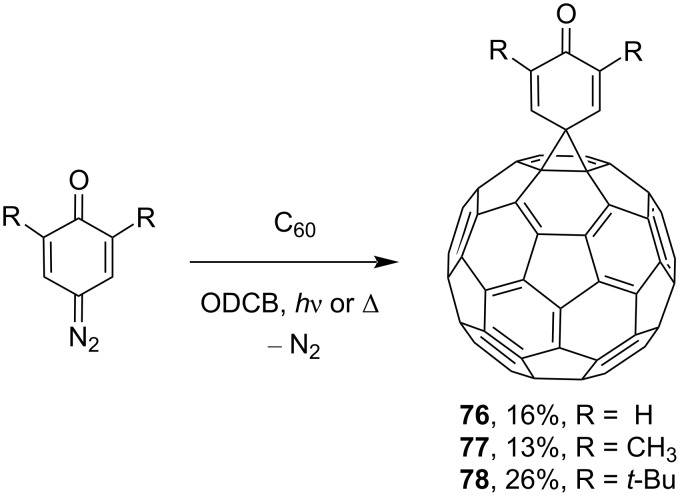
Synthesis of quinone-substituted methanofullerene derivatives **76**–**78**.

**Scheme 24:**
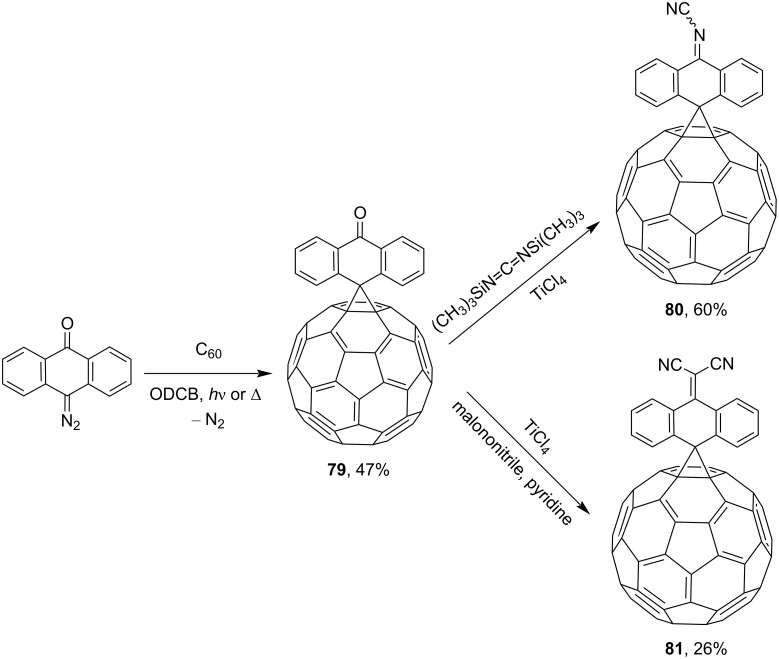
Synthesis of spiroannelated methanofullerenes **79**–**81**.

Some of the studies by Wudl and co-workers [108] deal with the synthesis of various spiromethanofullerenes. For example, spiro derivatives **73**–**75** were obtained in one of the first works on this subject (Figure 7). The authors obtained quinone-derived [6,6]-closed spiromethanofullerenes **76–78** by reactions of C_60_ with the corresponding diazo compounds upon heating, or photochemically in a nitrogen atmosphere (Scheme 23) [109]. The reaction of spiro[10-anthron-9,61'-methanofullerene] (**79**) [110] with bis(trimethylsilyl)carbodiimide and malononitrile in pyridine in the presence of TiCl_4_ resulted in spirocyclic blocks **80** and **81**, respectively (Scheme 24) [111].

**Figure 8:**
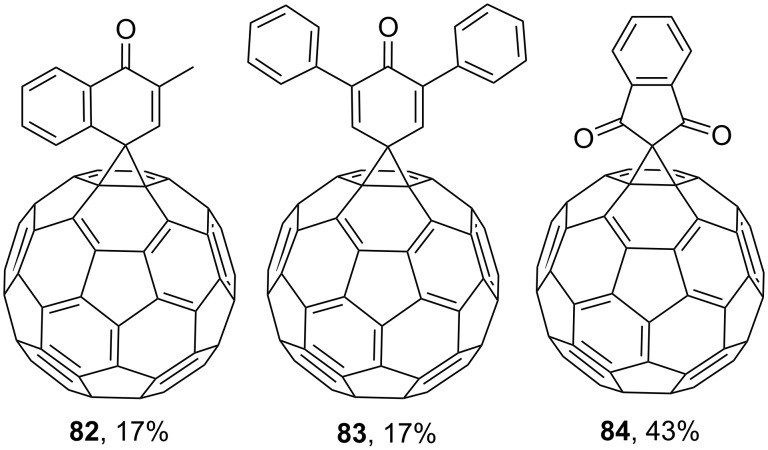
Quinone-substituted spiromethanofullerenes **82**–**84**.

Subsequently, the scope of quinone-substituted methanofullerenes was expanded by derivatives **82** and **83**, and the scope of indanedione-substituted derivatives by compound **84**. All of them were synthesized under conditions similar to those indicated in Scheme 24 (Figure 8) [112].

### Synthesis of methanofullerenes with tosylhydrazone

**Scheme 25:**
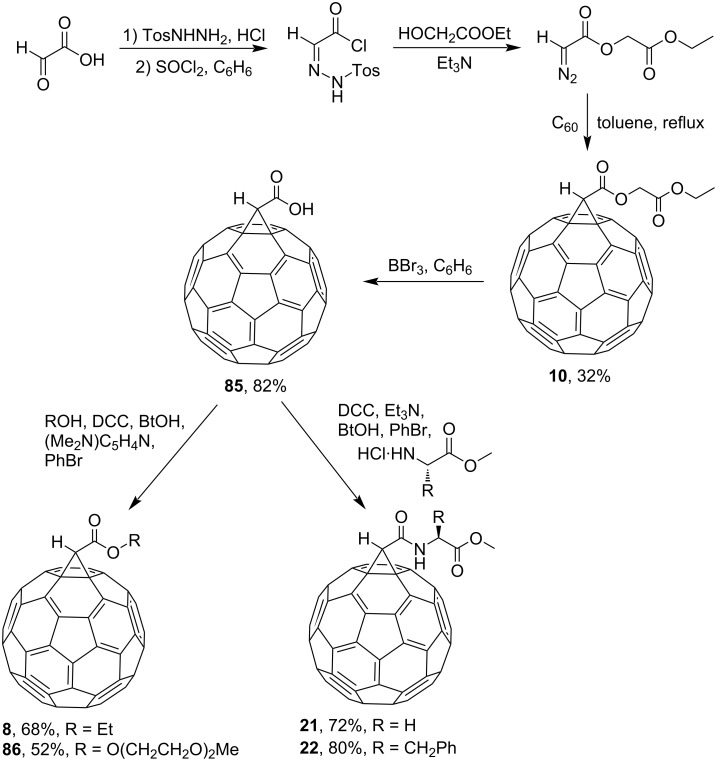
The synthetic route for methanofullerenes via tosyl derivatives. (BtOH is 1*H*-benzotriazol-1-ol.)

The first publication on the synthesis of methanofullerenes via tosyl derivatives appeared in 1993 [83]. According to the original source, C_60_ cyclopropanation is assumed to involve a diazo compound preliminarily synthesized from an aldehyde- or ketone-based tosylhydrazone. The authors aimed at finding a versatile approach for synthesizing methanofullerenes that would allow incorporating any functional group into the fullerene core: hydrophilic groups for biological tests and hydrophobic ones for photovoltaic studies. According to Reference [83], the intermediate methanofullerene-appended dicarboxylic acid **85** synthesized from **10** is a versatile synthon for conversion into numerous derivatives (Scheme 25). The reactivity of compound **85** was studied under DCC-promoted esterification conditions (see adducts **8** and **86**) and amidation conditions (see adducts **21** and **22**).

**Scheme 26:**
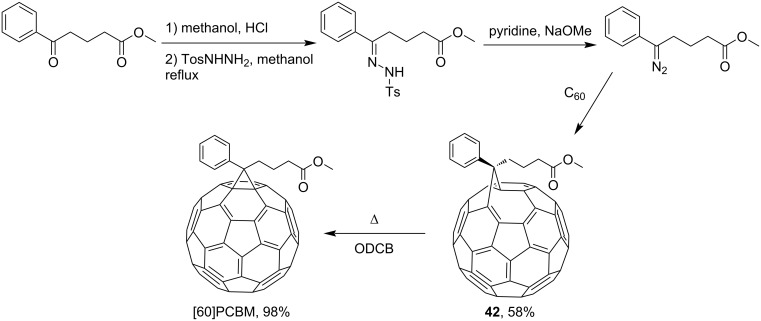
The synthetic route to [60]PCBM via tosylhydrazine.

Hummelen, Wudl, et al. [96] performed an optimized methanofullerene synthesis by carrying out the cyclopropanation in the one-pot version presented in reference [83]. Therein, fullerene was added directly to the solution of tosylhydrazone in the presence of a base. This procedure makes it possible to generate diazo compounds in situ, without the need for purification prior to the addition of C_60_. The process is successful even in the presence of unstable diazo compounds, and the yield of the target product increases significantly. The technique was tested in a synthesis of an acceptor component of organic solar cells, [60]PCBM, as an example (Scheme 26).

**Scheme 27:**
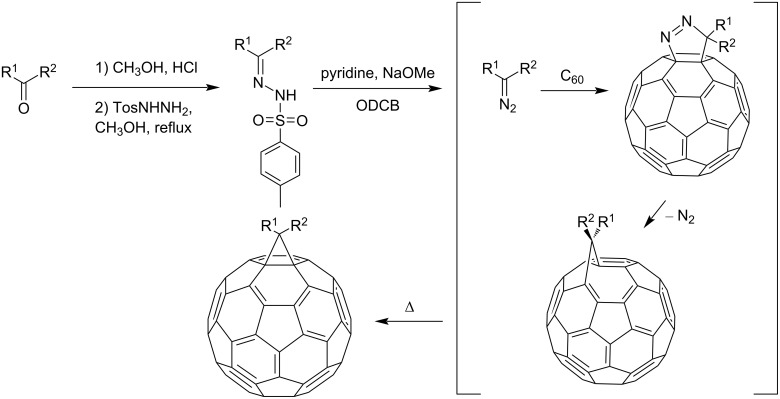
The reaction mechanism of fullerene cyclopropanation via tosylhydrazine derivatives.

The mechanism of fullerene cyclopropanation is presented in Scheme 27.

**Scheme 28:**
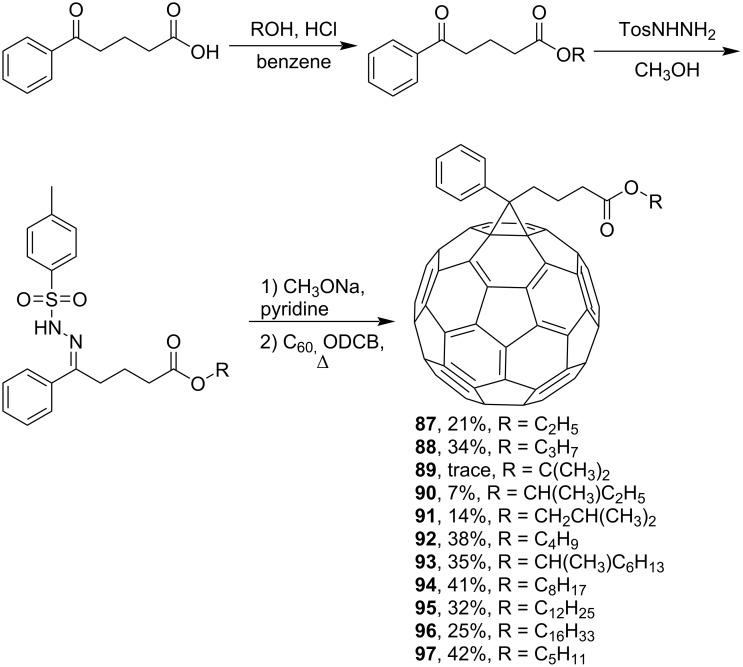
Serial synthesis of [6,6]-phenyl-C_61_-butyric acid esters **87**–**91** [114], **92**–**96** [113,115], and **97** [116].

Using a similar procedure, the reaction of C_60_ and alkyl-4-benzoyl butyrate *p-*tosylhydrazone in the presence of sodium methoxide and pyridine, the following [6,6]-phenyl-C_61_-butyric acid esters **87**–**97** with various alkyl-chain lengths were synthesized (Scheme 28). According to reference [113], the solubility of C_60_ derivatives in organic solvents increases with an increase in the length of alkyl substituents. The photovoltaic devices designed using poly(3-hexylthiophene)/methanofullerenes as the acceptor component, and compared to [60]PCBM, demonstrated that the derivative based on butyl ester **90** exhibited the best characteristics [113], but the best result in the study was obtained with the compound based on [60]PCBM methyl ester [63].

**Scheme 29:**
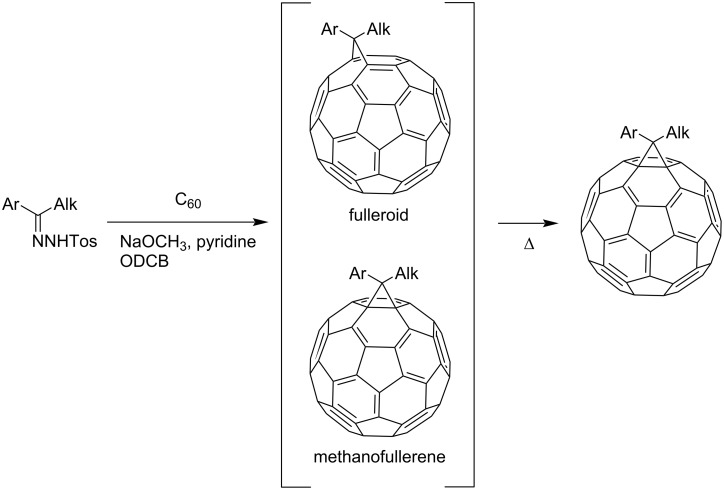
Typical procedure for a synthesis of [60]PCBM derivatives.

The next group of [60]PCBM derivatives synthesized by a similar technique and containing aromatic groups Ar on the one hand and alkyl groups Alk on the other, bound to the C_60_ core through a cyclopropane ring, most of which were tested in organic solar elements, is presented in Scheme 29, and the substituents are listed in Table 1 [117–140].

**Table 1:** [60]PCBM derivatives containing various aromatic and alkyl groups bound to the C_60_ core.

compound	Ar	Alk	yield (%)	Reference

**98**	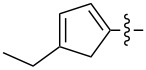	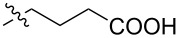	38	[117]
**99**	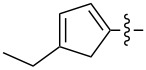	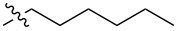	35	[117]
**100**	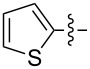	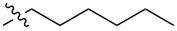	25	[118]
**101**	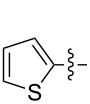	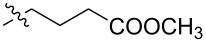	36, 53	[118–119]
**102**	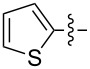	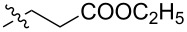	40	[120]
**103**	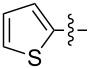	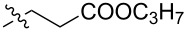	46	[120]
**104**	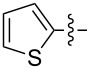	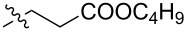	50	[120]
**105**	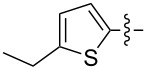	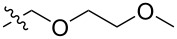	43	[120]
**106**	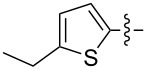	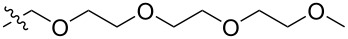	42	[120]
**107**	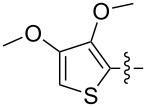	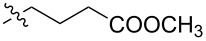	72	[119]
**108**	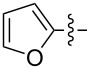	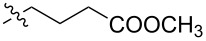	20	[118]
**109**	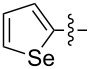	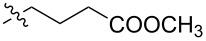	20	[121]
**110**	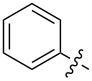	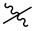	50	[79]
**111**	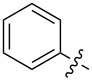	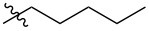	nr^a^	[122]
**112**	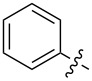	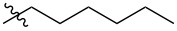	nr	[122]
**113**	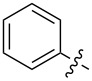	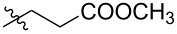	nr	[123–124]
**114**	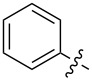	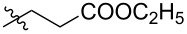	nr	[123]
**115**	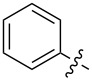	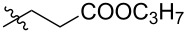	nr	[123]
**116**	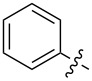	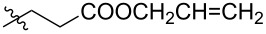	40	[125]
**117**	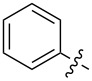	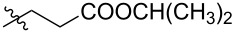	nr	[123]
**118**	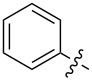	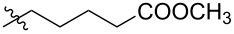	nr	[124]
**119**	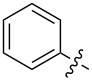	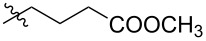	nr	[124]
**120**	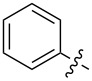	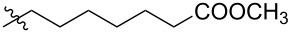	nr	[124]
**121**	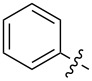	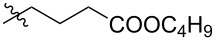	nr	[123]
**122**	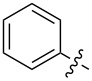	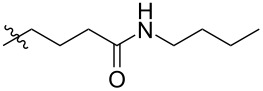	nr	[126]
**123**	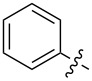	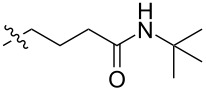	nr	[126]
**124**	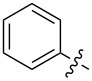	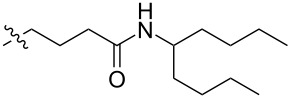	30	[127]
**125**	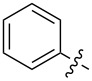	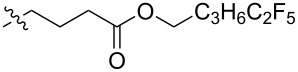	nr	[128]
**126**	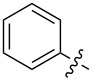	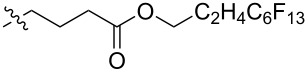	nr	[128]
**127**	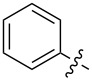	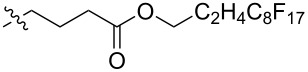	nr	[128]
**128**	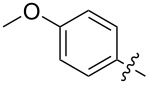	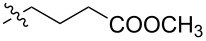	nr	[122]
**28**	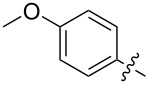	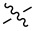	42	[79]
**129**	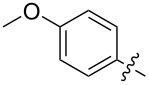	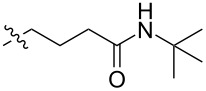	nr	[126]
**130**	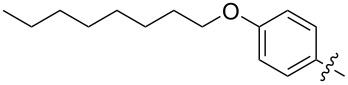	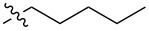	17	[129]
**131**	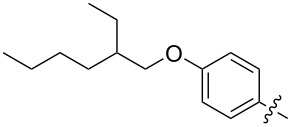	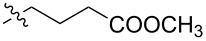	34	[130]
**132**	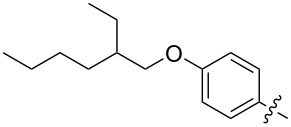	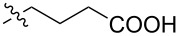	96	[131]
**133**	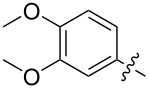	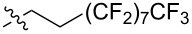	nr	[132]
**134**	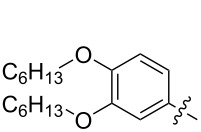	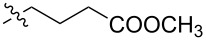	7.5	[133]
**135**	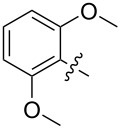	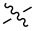	40	[79]
**136**	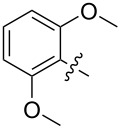	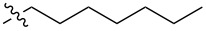	50	[132]
**137**	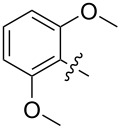	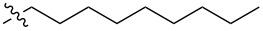	51	[132]
**138**	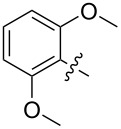	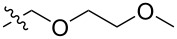	43	[132]
**139**	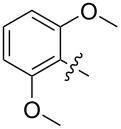	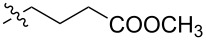	44	[132]
**140**	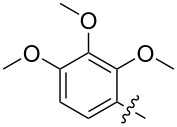	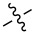	35	[132]
**141**	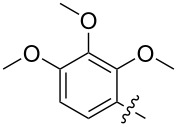	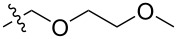	28	[132]
**142**	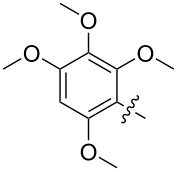	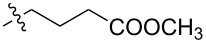	49	[133]
**143**	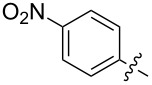	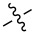	37	[79]
**144**	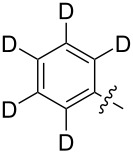	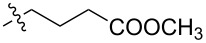	39	[134]
**145**	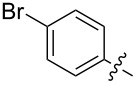	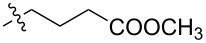	47	[134]
**146**	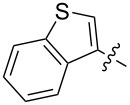	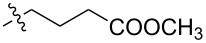	46	[135]
**147**	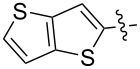	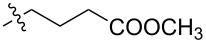	50	[135]
**148**	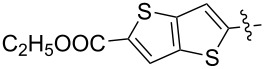	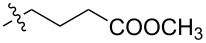	44	[135]
**149**	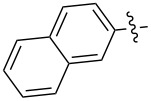	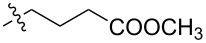	27	[136]
**150**	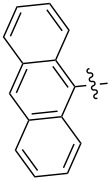	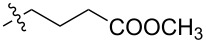	11	[136]
**151**	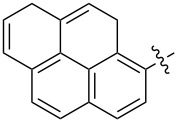	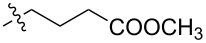	22	[136]
**152**	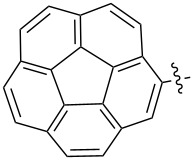	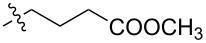	41	[136]
**153**	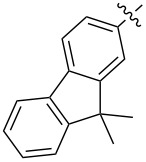	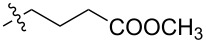	33	[137]
**154**	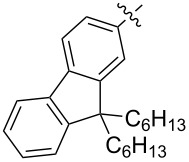	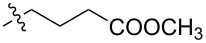	42	[138]
**155**	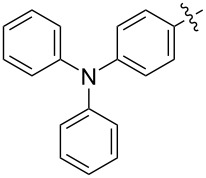	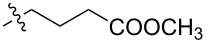	35	[137]
**156**	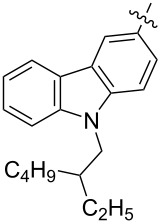	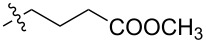	18	[139]
**157**	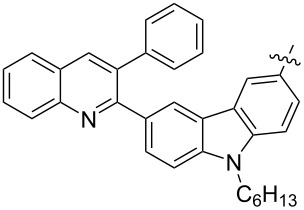	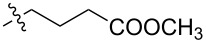	52	[140]
**158**	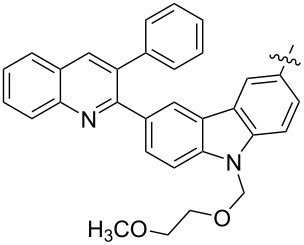	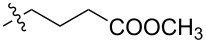	49	[140]

^a^The yield has not been reported.

**Figure 9:**
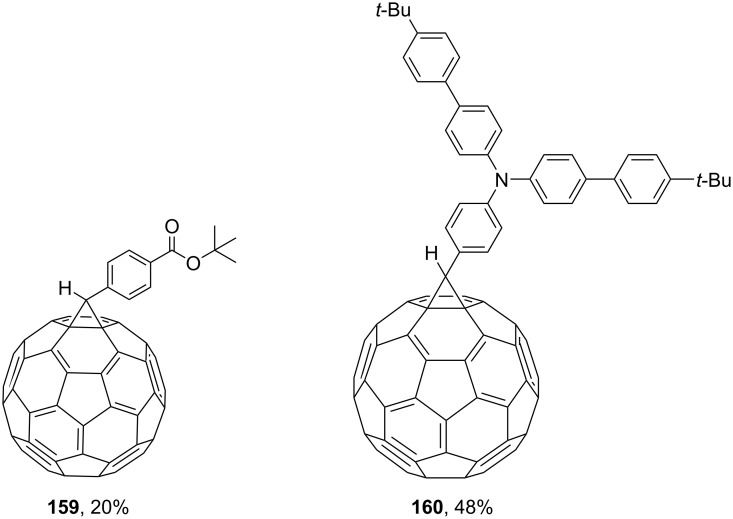
Structures of 4-C_61_-phenylorganofullerenes **159** and **160**.

**Figure 10:**
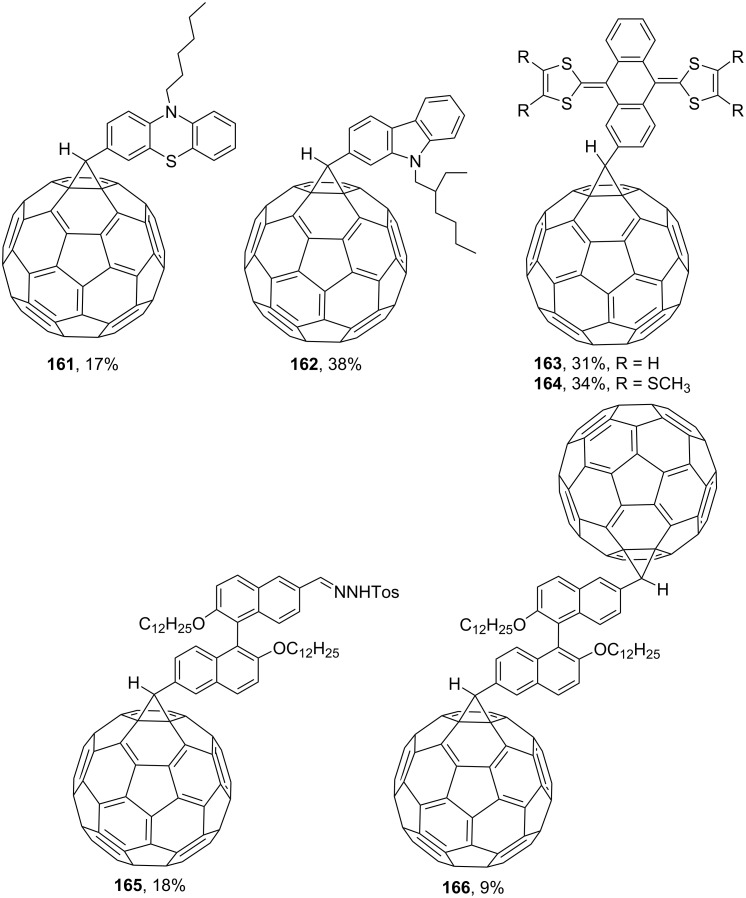
Structures of methanofullerenes containing aromatic polycyclic systems.

The tosylhydrazone method discussed above was used to obtain monoaryl derivatives, such as the aforementioned **29** [79], **30** (in 55% yield) [141] as well as **159** [142] and **160** (Figure 9) [143]. Representatives containing polycyclic aromatic systems based on phenothiazine **161** [144], carbazole **162** [145], and tetrathiofulvalenes **163** and **164** [146] and 1,1-binaphthalenes **165** and **166** [147] are known. They are shown in Figure 10.

**Figure 11:**
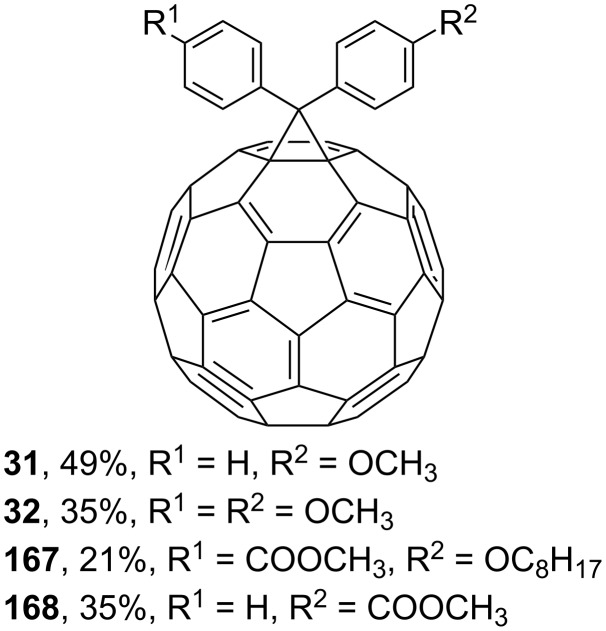
4,4-Diphenylfullerene derivatives **31**, **32**, **167**, and **168**.

**Figure 12:**
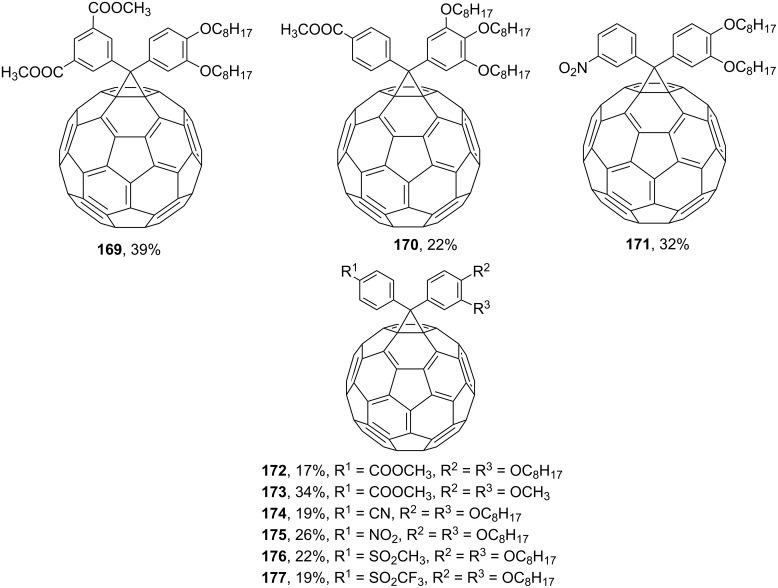
Polysubstituted diphenylmethanofullerenes **169**–**177**.

Various diaryl derivatives of C_60_ can also be obtained by this approach. Thus, the structures of 4,4'-diphenylfullerene derivatives synthesized are shown in Figure 11, and a number of diphenylmethanofullerenes **169**–**177** with electron-withdrawing groups are reported in reference [148] (Figure 12).

**Scheme 30:**
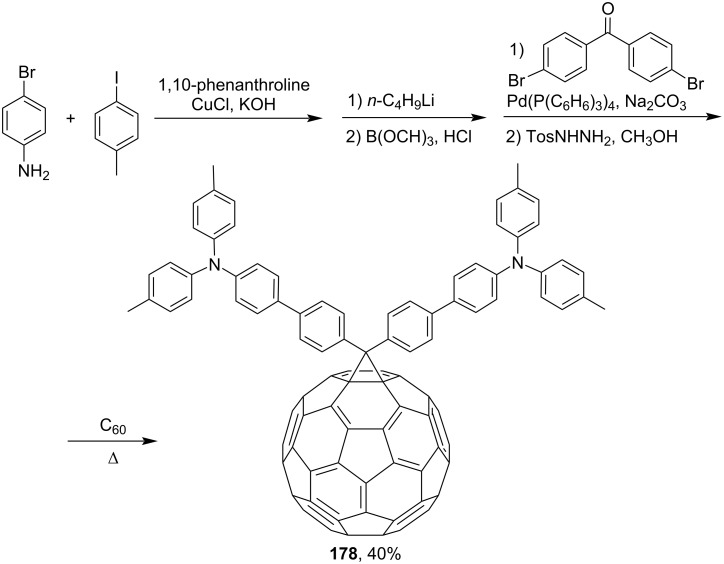
The synthetic route to triphenylamine-substituted methanofullerene **178**.

**Figure 13:**
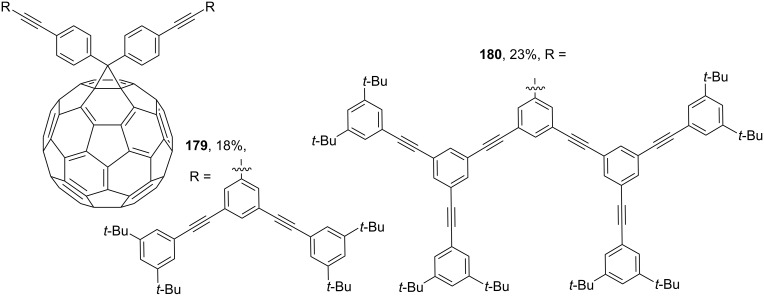
Fullerene-containing phenylacetylene dendrimers of the 1st and 2nd generation.

Publication [149] describes compound **178** containing triphenylamine donor groups (Scheme 30). An interesting approach was used in reference [150], and the syntheses of phenylacetylene dendrimers of the 1st (see **179**) and 2nd generation (see **180**) are reported (Figure 13).

**Scheme 31:**
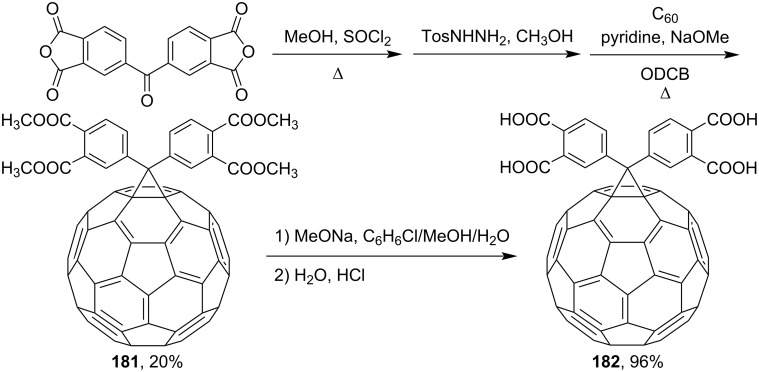
The synthetic route to water-soluble methano[60]fullerene-appended tetracarboxylic acid derivatives.

In reference [151], the reaction of fullerene with 3.3',4.4'-tetrakis(methoxycarbonyl)benzophenone tosylhydrazone first gave ester **181** and then acid **182** (Scheme 31), the sodium and potassium salts of which are well soluble in water. The compound has antiviral and anticancer activity as well as pronounced antioxidant properties combined with low toxicity.

**Scheme 32:**
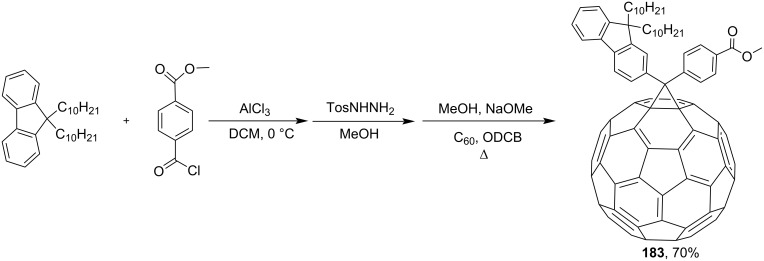
The synthetic route to diarylmethanofullerene **183**.

Diarylmethanofullerene **183** was synthesized according to Scheme 32 from 9,9-didecyl-9*H*-fluorene and monomethyl terephthalate chloride [152].

**Scheme 33:**
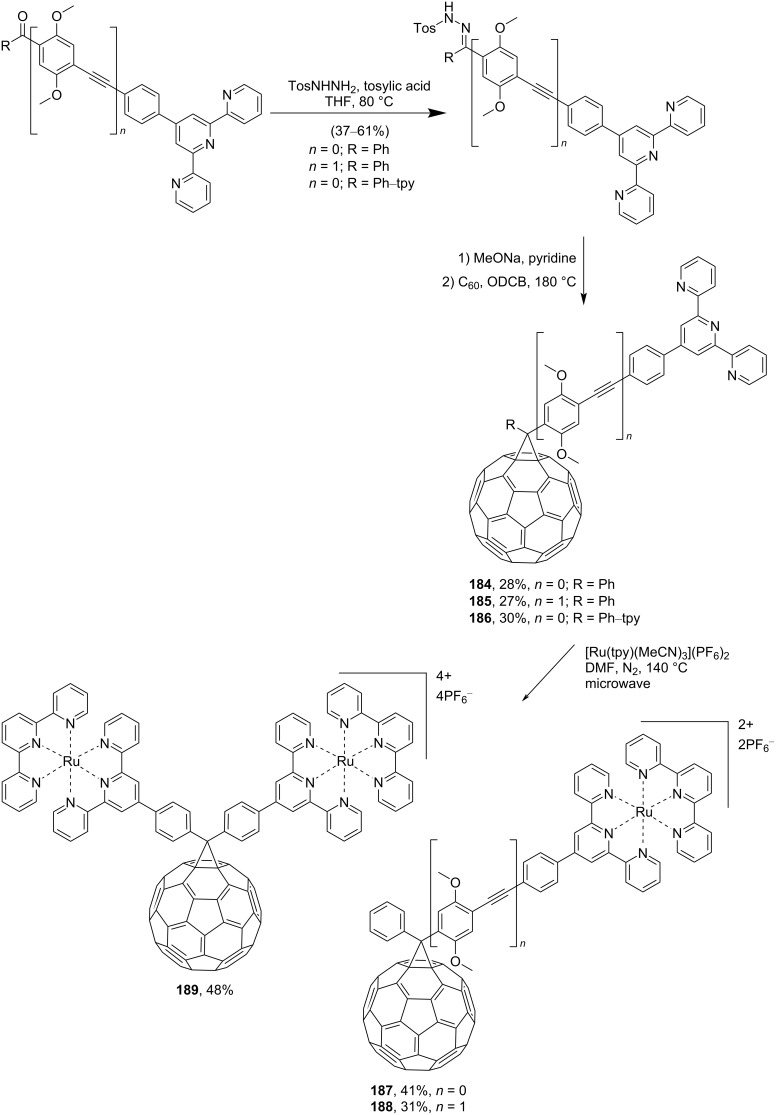
The synthetic route to tpy-containing methanofullerene dyads **184**–**186**, coordination of which with ruthenium(II) gives donor–bridge–acceptor assemblies **187**–**189**.

The authors of reference [153] presented a series of 2,2';6',2"-terpyridine (tpy)-substituted methanofullerenes and pyrrolidinofullerene dyads **184**–**186** linked through *para*-phenylene or *para*-phenyleneethynylphenylene units (Scheme 33). Сoordination of these compounds with ruthenium(II) leads to a donor–bridge–acceptor assembly of complexes with various lengths, **187**–**189**. It is believed [153] that the photophysical and electrochemical properties of the complexes presented are promising for the formation of charge-separated photoinduced states for artificial photosynthetic devices, in particular where assemblies are expanded from dyads to triads by including a lateral organic or organometallic donor.

**Scheme 34:**
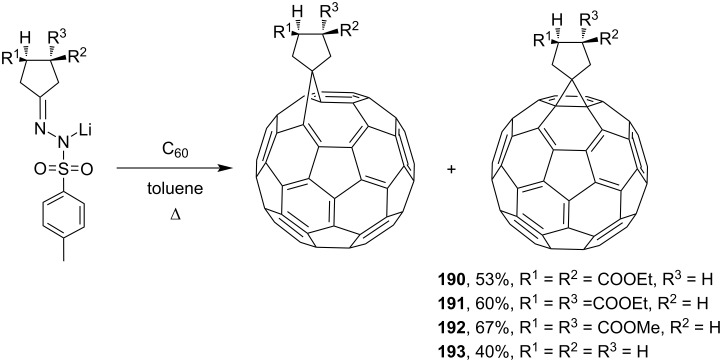
Synthesis of a series of spirocyclopentalydenemethanofullerenes **190**–**193**.

The synthesis of a whole series of spiromethanofullerenes was carried out through the stage where tosylhydrazone derivatives were formed. In fact, a series of spirocyclopentalydenemethanofullerenes **190**–**193** were obtained by the reaction of C_60_ with lithium salts of tosylhydrazone (Scheme 34) [154].

**Scheme 35:**
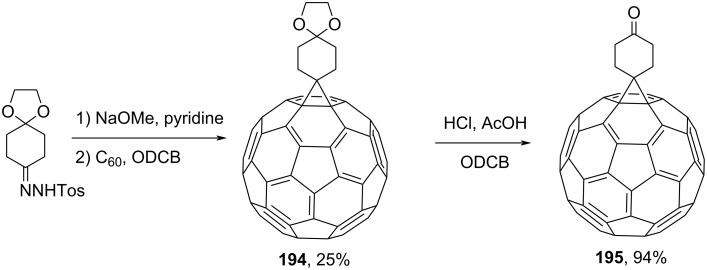
Synthesis of spiromethanofullerenes **194** and **195**.

**Figure 14:**
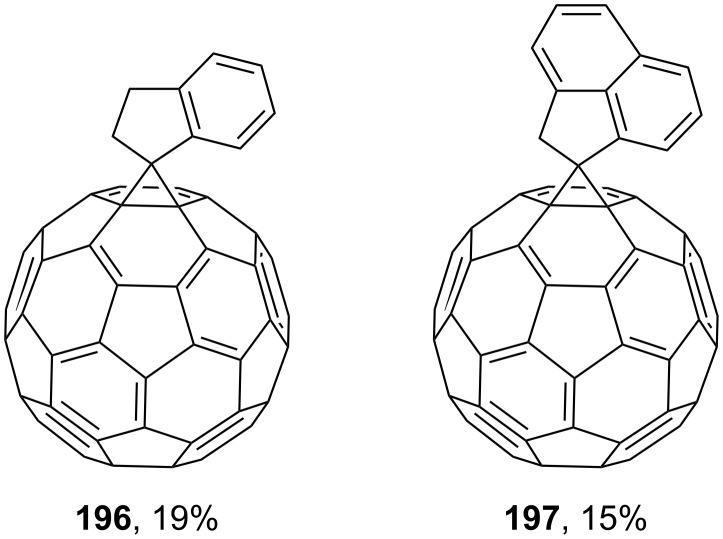
Dibenzosuberane-substituted fullerene derivatives **196** and **197**.

Spiromethanofullerene **194** was prepared on the basis of monoethylene glycol tosylhydrazone. Hydrolysis of the former resulted in 6,6-spiro[cyclohexanone-4,61'-methanofullerene] (**195)** (Scheme 35) [111]. Fullerene-containing adducts such as benzocyclopentanefullerene **196**, acenaphthenefullerene **197** [155], and dibenzosuberanefullerene **73** [156] are also known (Figure 14).

**Scheme 36:**
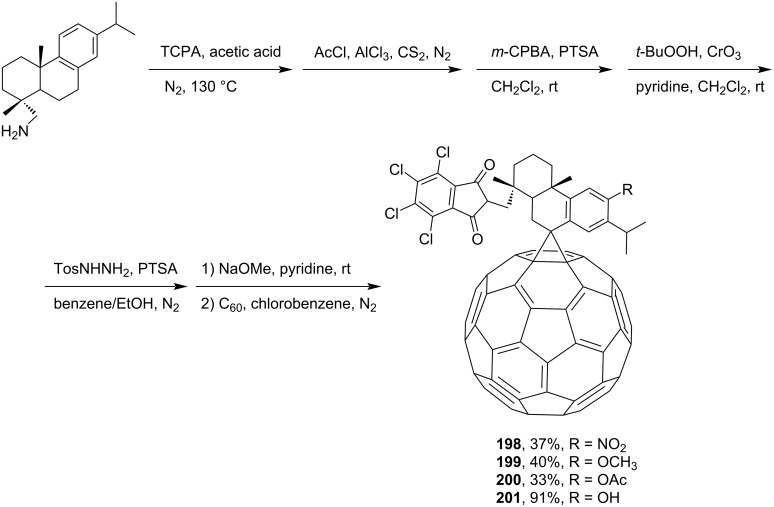
The synthetic route to ring-B-C_60_-attached derivatives of *N*,*N*-(tetrachlorophthaloyl)dehydroabietylamine, **198**–**201**.

Unusual spiromethanofullerenes were presented in reference [157], where the synthesis of *N*,*N*-(tetrachlorophthaloyl)dehydroabietylamine derivatives **198**–**201** from dehydroabietylamine substituted by fullerene in ring B was reported (Scheme 36). These compounds were obtained in a search for new derivatives of rosin amines with potential biological activity.

**Scheme 37:**
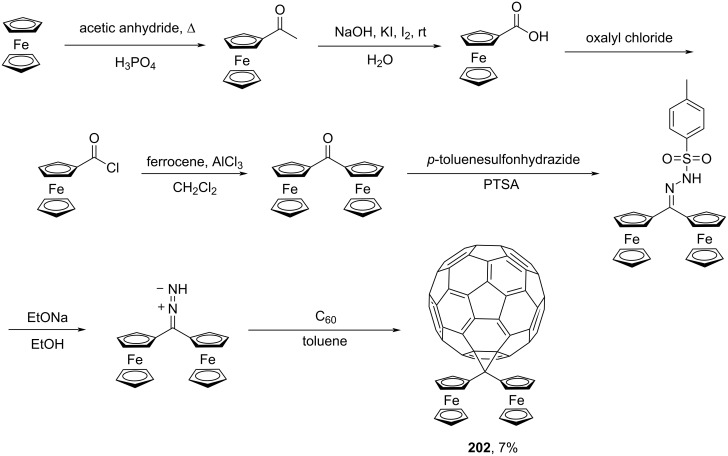
The synthetic route to methano-bridged diferrocenylfullerene **202**.

Further, an unusual ferrocene-containing block **202** was synthesized (Scheme 37) [158].

**Scheme 38:**
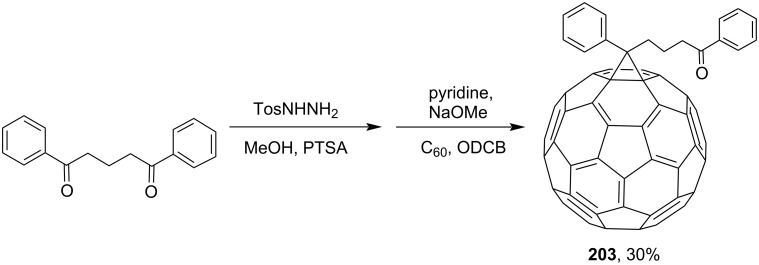
The synthetic route to 1-(3-(benzoyl)propyl)-1-phenyl[6,6]-C_60_ **203**.

**Scheme 39:**
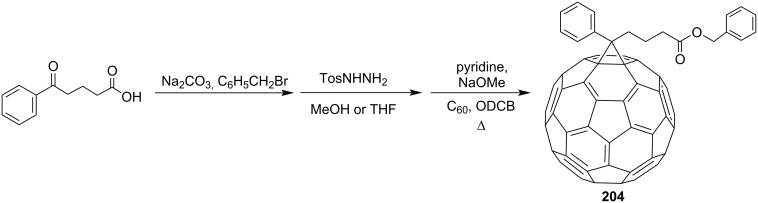
The synthetic route to methanofullerene **204**.

**Scheme 40:**
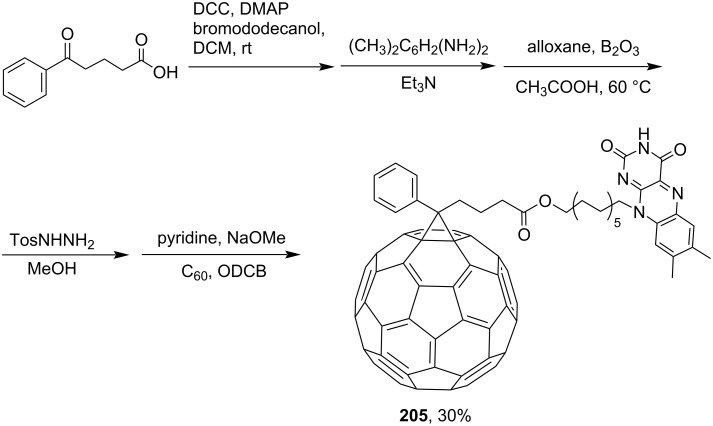
The synthetic route to C_60_-functionalized flavin **205**.

1-(3-(Benzoyl)propyl)-1-phenyl[6,6]methanofullerene (**203**) was obtained by 1,3-dipolar cycloaddition of a tosylhydrazone derivative (obtained from 1,3-dibenzoylpropane) to C_60_ (Scheme 38) [159]. 2-Benzoylpropionic acid was used as the starting material for synthesizing adduct **204** (Scheme 39) [123]. A synthesis and a self-assembly of flavin-functionalized fullerene derivative **205** consisting of [60]PCBM and isoalloxazine moieties attached at both ends of a linear 12-carbon aliphatic spacer, based on the same acid (Scheme 40), was reported [160].

**Scheme 41:**
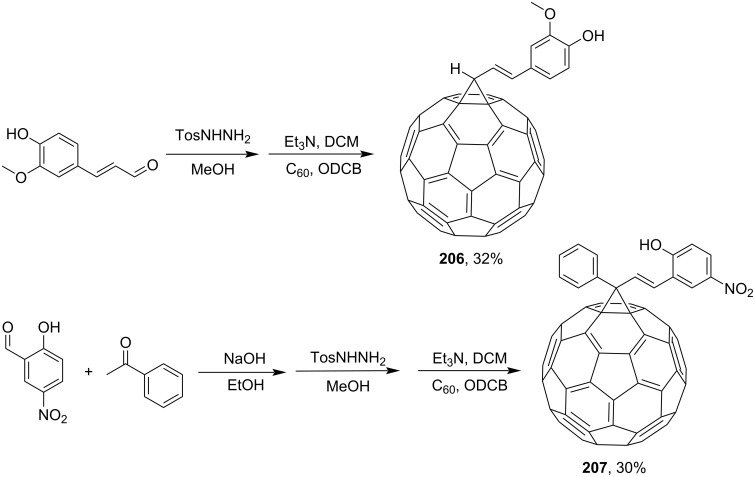
The synthetic route to conjugates of C_60_ with cinnamaldehyde **206** and with chalcone **207**.

**Scheme 42:**
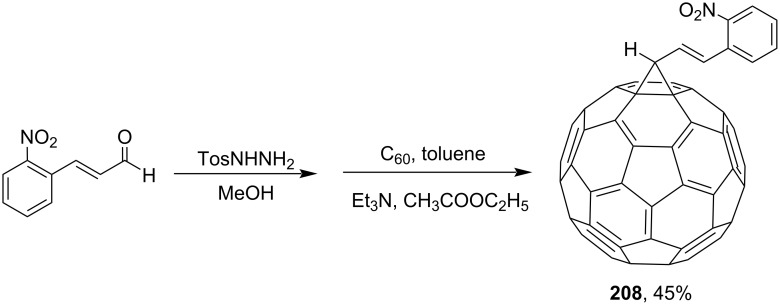
The synthetic route to conjugate C_60_ with a 2-nitrocinnamyl group.

In a study by Kumar and co-workers [161], the synthesis of methanofullerenes **206** and **207** was based on 3-methoxy-4-hydroxycinnamic aldehyde and a chalcon obtained by Claison condensation of acetophenone with 2-hydroxy-5-nitrobenzaldehyde (Scheme 41). Further, the same team obtained methanofullerene adduct **208** based on 2-nitrocinnamic aldehyde (Scheme 42) [116].

**Scheme 43:**
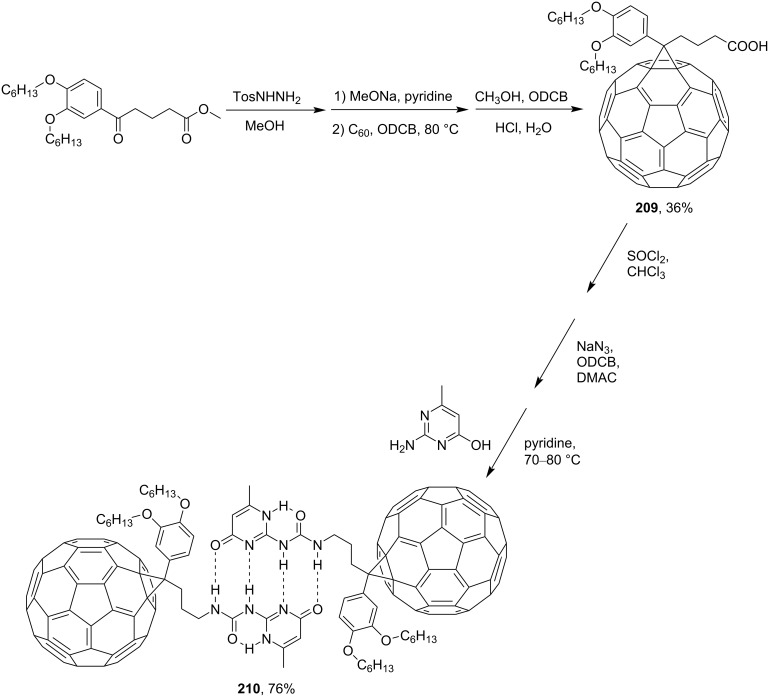
The synthetic route to a C_60_ dimer connected through a highly directional fourfold hydrogen bonding motif.

Hummelen and co-workers [131] worked on creating derivatives of H-bound fullerene associates. For instance, methyl 5-oxo-5-(3,4-bishexyloxy)phenylpentanoate was converted via a tosyl derivative to a methanofullerene derivative of butyric acid **209**. Based on this, fullerene dimer **210** with four hydrogen bonds having a very high dimerization constant was obtained (Scheme 43).

**Scheme 44:**
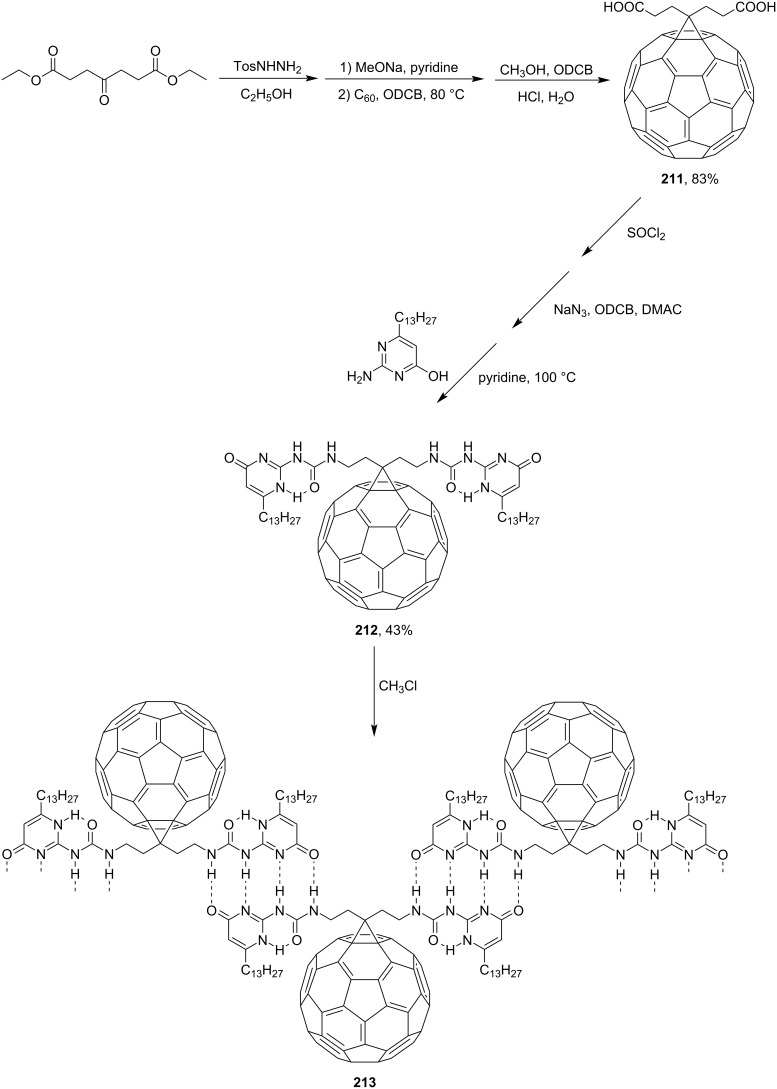
The synthetic route to quadruple hydrogen‐bonded fullerene array **213**.

Diethyl 4-oxopimelate was used as the precursor of polymer **213**. The former was converted via diacid **211** to the target methanofullerene **212**, the solution of which in chloroform shows typical resonance signals of six hydrogen-bonded protons (Scheme 44) [162].

**Scheme 45:**
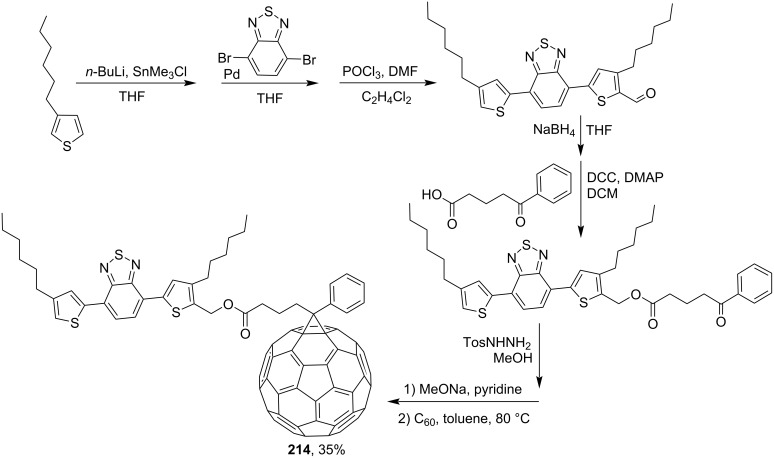
The synthetic route to conjugates **214** of fullerene with benzothiadiazole.

**Scheme 46:**
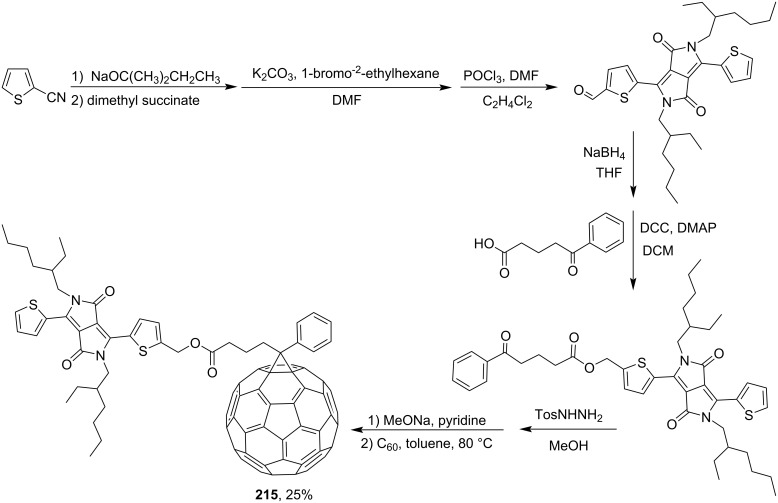
The synthetic route to conjugates **215** of fullerene with diketopyrrolopyrrole.

In order to improve the optical absorption of fullerene acceptors for utilization in solar cells with a bulk heterojunction, a series of dye–fullerene dyads with strong absorption in the visible region was synthesized [163]. A synthesis of C_60_ fullerene conjugate **214** with a benzothiadiazole moiety is shown in Scheme 45, while Scheme 46 presents a synthetic pathway to diketopyrrolopyrrole-modified conjugate **215**.

**Scheme 47:**
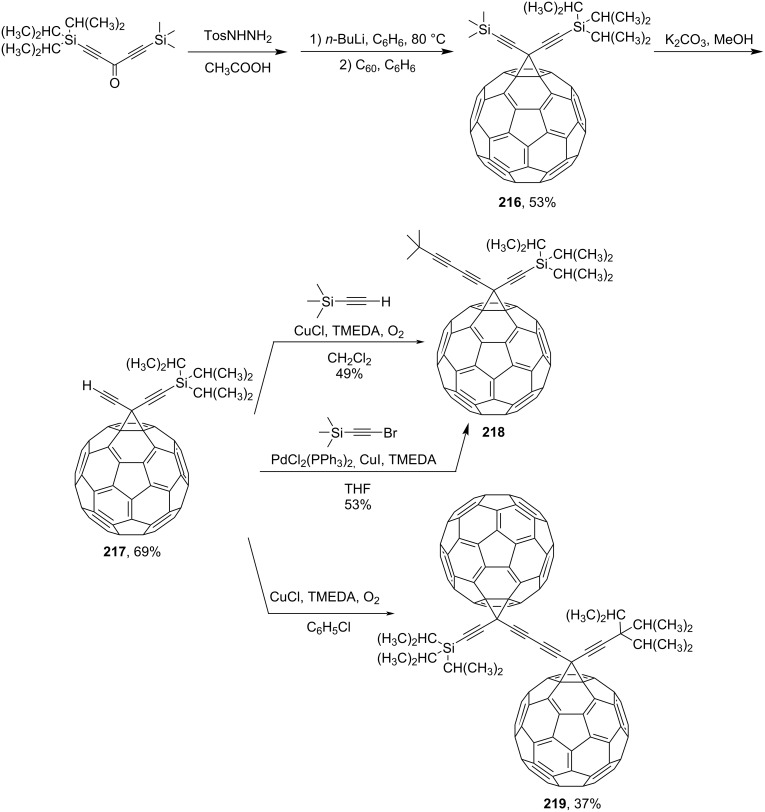
The synthetic route to fullerene–acetylene hybrids.

Other methanofullerenes without aromatic groups obtained by this method are presented below. Fullerene–acetylene derivatives **218** and **219** obtained by the tosylhydrazone method (Scheme 47) are also reported in the literature [164–165]. First, orthogonally protected diethinylmethanofullerene **216** was obtained and then converted to **217** by protodesilylation. Finally, blocks **218** and **219** were obtained by oxidative hetero- and homocoupling of **217**, respectively. It is also noted that dumbbell-like fullerene-containing compound **219** is the first example of a dimeric fullerene that was fully characterized.

## Conclusion

Summarizing our analysis of scientific literature on [2 + 1] cycloaddition reactions to C_60_ fullerene based on diazo compounds allows us to state the following: An alternative to the synthesis of methanofullerenes by the Bingel methodology is provided by the thermal [2 + 1] cycloaddition of diazo compounds to the fullerene. Then follows the photolysis or thermolysis of pyrazoline intermediates by N_2_ extrusion. Therein, the formed [5,6]-opened adducts are transformed into [6,6]-closed isomers, which are more thermodynamically stable. While the range of reagents used in nucleophilic cyclopropanation with stabilized carbanions is mainly limited to malonates, the range of cyclopropanating agents involved in C_60_ reactions with diazo compounds is much wider. In addition, the high yield of the target products and the nearly unlimited variability of substituents in the cyclopropane moiety should be noted as advantages of the latter method. Nevertheless, a drawback that all the [2 + 1] cycloaddition reactions to fullerene have in common is that, due to functionalization of the C_60_ core, formation of a mixture of hardly separable polyaddition products is observed, rather than selective formation of a certain compound. It is the reason why the search for methods for controlling the C_60_ cyclopropanation process is so relevant.

The scope of the fields of use of fullerenes discussed in the scientific literature is almost unlimited, while the synthesis of derivatives based thereon and the subsequent studies expand the borders of the application areas. Taking all of the above into consideration, it can be assumed that the fruitful development of the chemistry of fullerene derivatives will continue for many years to come. We believe that information presented herein will be useful to synthetic chemists for analyzing their achievements and for choosing their own areas of research in this area.
